# Tom20 gates PINK1 activity and mediates its tethering of the TOM and TIM23 translocases upon mitochondrial stress

**DOI:** 10.1073/pnas.2313540121

**Published:** 2024-02-28

**Authors:** Mohamed A. Eldeeb, Andrew N. Bayne, Armaan Fallahi, Thomas Goiran, Emma J. MacDougall, Andrea Soumbasis, Cornelia E. Zorca, Jace-Jones Tabah, Rhalena A. Thomas, Nathan Karpilovsky, Meghna Mathur, Thomas M. Durcan, Jean-François Trempe, Edward A. Fon

**Affiliations:** ^a^McGill Parkinson Program and Neurodegenerative Disorders Research Group, Department of Neurology and Neurosurgery, Montreal Neurological Institute-Hospital, McGill University, Montréal, QC H3A 2B4, Canada; ^b^Structural Genomics Consortium - Neuro, McGill University, Montréal, QC H3A 2B4, Canada; ^c^Department of Pharmacology and Therapeutics, McGill University, Montréal, QC H3G 1Y6, Canada; ^d^Centre de Recherche en Biologie Structurale, Montréal, QC H3G 0B1, Canada

**Keywords:** mitochondrial quality control, PINK1, mitophagy, mitochondrial import, proteolysis

## Abstract

Mutations in PTEN-induced putative kinase 1 (PINK1) cause autosomal recessive early-onset Parkinson’s disease (PD). PINK1 is a Ser/Thr kinase that regulates mitochondrial quality control by triggering mitophagy mediated by the ubiquitin (Ub) ligase Parkin. Upon mitochondrial damage, PINK1 accumulates on the outer mitochondrial membrane forming a high-molecular-weight complex with the translocase of the outer membrane (TOM). PINK1 then phosphorylates Ub, which enables recruitment and activation of Parkin followed by autophagic clearance of the damaged mitochondrion. Thus, Parkin-dependent mitophagy hinges on the stable accumulation of PINK1 on the TOM complex. Yet, the mechanism linking mitochondrial stressors to PINK1 accumulation and whether the translocases of the inner membrane (TIMs) are also involved remain unclear. Herein, we demonstrate that mitochondrial stress induces the formation of a PINK1-TOM-TIM23 supercomplex in human cultured cell lines, dopamine neurons, and midbrain organoids. Moreover, we show that PINK1 is required to stably tether the TOM to TIM23 complexes in response to stress such that the supercomplex fails to accumulate in cells lacking PINK1. This tethering is dependent on an interaction between the PINK1 N-terminal–C-terminal extension module and the cytosolic domain of the Tom20 subunit of the TOM complex, the disruption of which, by either designer or PD-associated PINK1 mutations, inhibits downstream mitophagy. Together, the findings provide key insight into how PINK1 interfaces with the mitochondrial import machinery, with important implications for the mechanisms of mitochondrial quality control and PD pathogenesis.

## Significance

Loss-of-function mutations in PTEN-induced putative kinase 1 (PINK1) cause Parkinson’s disease through physiological processes that have yet to be fully elucidated. PINK1 is a kinase that accumulates selectively on damaged mitochondria, where it recruits the E3 ubiquitin ligase Parkin to mediate mitophagy. This cascade hinges on PINK1 binding to the translocase of the outer membrane (TOM) complex; however, the determinants of PINK1-TOM complex assembly remain elusive. Herein, we investigate the formation of a PINK1-TOM-TIM23 supercomplex under various stressors, characterize the subunit composition of this supercomplex, and highlight a key interaction between PINK1 and Tom20 which gates downstream PINK1 activity. Overall, these findings provide insight into how PINK1 interfaces with the mitochondrial import machinery to regulate mitophagy.

Parkinson’s disease (PD) is a progressive neurodegenerative condition characterized by the selective loss of dopaminergic neurons in the substantia nigra of the midbrain (1). Importantly, current treatments only address the symptoms caused by the ensuing dopamine (DA) deficiency but not the underlying molecular mechanisms that lead to neurodegeneration (1, 2). Analyses of the genes that cause inherited forms of PD point to mitochondrial dysfunction as a major contributor to the etiology of PD (2–4). An inherent challenge that mitochondria continuously encounter is the exposure to diverse stresses including high levels of reactive oxygen species and protein misfolding, which increase their likelihood of dysfunction (2, 5, 6). In response, eukaryotic cells have evolved an elaborate series of quality control mechanisms to identify, repair, and/or eliminate defective mitochondria (7–11). One such mechanism is PTEN-induced putative kinase 1 (PINK1)/Parkin mitophagy (selective degradation of mitochondria by autophagy), a process which involves PINK1, a mitochondrial Ser/Thr kinase (12), and Parkin, an E3 ubiquitin (Ub) ligase, encoded respectively by *PINK1* and *PRKN*, two genes in which loss-of-function mutations cause autosomal recessive early-onset PD (2, 12, 13). PINK1 acts upstream of Parkin and is essential for the mitochondrial localization and activation of Parkin. Upon mitochondrial damage, PINK1 builds up on the outer mitochondrial membrane (OMM) (14–21) where it phosphorylates both Ub and the Ub-like domain of Parkin (17, 22). Activated Parkin then ubiquitinates numerous OMM proteins, notably Mfn2 and VDAC (23–25), to initiate mitophagy (21, 22, 26). Thus, PINK1 acts a damage sensor on mitochondria, orchestrating the clearance of unhealthy mitochondria by Parkin (2, 21, 22, 27).

The PINK1 protein consists of an amino-terminal mitochondrial targeting sequence, a transmembrane helix, and a 52-kDa cytosolic kinase domain. The cytosolic fragment alone carries Ub kinase activity and bears features of protein kinases such as catalytic and activation loops (21, 22, 28). PINK1 mRNA is cotransported with mitochondria in neurons, and the translated PINK1 protein is imported through the translocase of the outer membrane (TOM) complex, a large multimeric channel that is critical for the import of mitochondrial precursor proteins (2, 21, 29). Following import, PINK1 is cleaved at its N terminus by MPP in the matrix and PARL in the inner mitochondrial membrane (IMM) (30, 31). While the mechanisms of PINK1 translocation to the PARL active site are still unknown, it has been proposed that AFG3L2, an IMM AAA+ protease, may play a role in the membrane dislocation of the PINK1 transmembrane domain (TMD) (a.a. 94 to 110) into a PARL-competent orientation (30, 32). Upon PARL cleavage between A103 and F104, PINK1 is retro-translocated to the cytosol where it is rapidly degraded by the proteasome-dependent N-degron degradation machinery (21, 32, 33). Thus, at steady-state, PINK1 levels are kept low, as corroborated by PINK1 half-life measurements around 30 min (34, 35). Remarkably, upon mitochondrial depolarization and import failure, commonly induced by treatment of cells with the protonophore cyanide m-chlorophenyl hydrazone (CCCP), PINK1 is no longer cleaved by proteases and builds up as a high-molecular-weight (HMW) 720- to 800-kDa complex associated with the TOM machinery at the OMM (18, 19). The stabilization of PINK1 hinges on a motif of negatively charged glutamic acid residues (3E), E112, E113, and E117, at the beginning of the N-terminal (NT) helix located between the TMD and the kinase domain, as well as the Tom7 subunit of the TOM complex (32, 36). Our group solved the crystal structure of the entire cytosolic domain of PINK1, revealing that the NT helix forms a module with its C-terminal extension (CTE), which is critical for PINK1 stabilization on TOM and subsequent activation by *trans* autophosphorylation on Ser228 (37). The NT-CTE module harbors PD mutations, which impair PINK1 activation and Parkin recruitment to mitochondria (32, 37, 38), thus highlighting the importance of characterizing this module.

The determinants for PINK1 import and accumulation at the IMM remain less defined: While most matrix-destined mitochondrial proteins rely on the translocase of the inner membrane (TIM23) complex and matrix import motor (primarily Tim44 and mtHsp70), there have been conflicting results on whether Tim23 knockdown affects PINK1 stabilization, PARL cleavage, and/or PINK1-TOM complex formation (32, 39). In one report, knockdown of Tim23 had no effect on CCCP-dependent PINK1 accumulation (32), while in another it reduced both PINK1 accumulation and PINK1-TOM complex assembly (39). While it has been shown that TIM23-dependent substrates are typically transferred upon exiting the Tom40 pore to Tim50 [the primary receptor of the TIM23 complex which contains a presequence binding domain exposed to the intermembrane space (IMS)] (40–44), the potential interplay between PINK1 and Tim50 remains poorly characterized. It has also been reported that the IMM metalloprotease OMA1 can cleave PINK1 in depolarized or Tom7 knockout (KO) mitochondria to degrade misassembled PINK1 and prevent its aberrant hyperaccumulation (32, 39). Therefore, the mechanism of the PINK1 association with the mitochondrial protein import machinery needs to be further elucidated.

Herein, we demonstrate that in response to two distinct mitochondrial stressors, depolarization and misfolded proteins, PINK1 forms a supercomplex with the TOM and TIM23 complexes in multiple human cell types, including PD-relevant induced pluripotent stem cell (iPSC)-derived DA neurons and midbrain organoids. Using affinity purification-mass spectrometry in combination with AlphaFold, we identify an interaction between the NT-CTE module of PINK1 and the Tom20 subunit of the TOM complex, which we show is required for PINK1-TOM-TIM23 supercomplex assembly, PINK1 kinase activation, and downstream Parkin-mediated mitophagy. Importantly, PD-associated PINK1 mutations within this NT-CTE-Tom20 interface interfere with PINK1 supercomplex assembly and downstream mitophagy. Finally, we show that in the absence of PINK1, TOM, and TIM23 fail to assemble into a stable supercomplex. Thus, our work positions PINK1 as an endogenous import substrate required to tether the TOM and TIM23 complexes into a stable supercomplex in response to mitochondrial stress in mammalian cells.

## Results and Discussion

### PINK1 Assembles into a HMW Complex upon Mitochondrial Depolarization in Human DA Neurons and Midbrain Organoids.

**Fig. 1.**
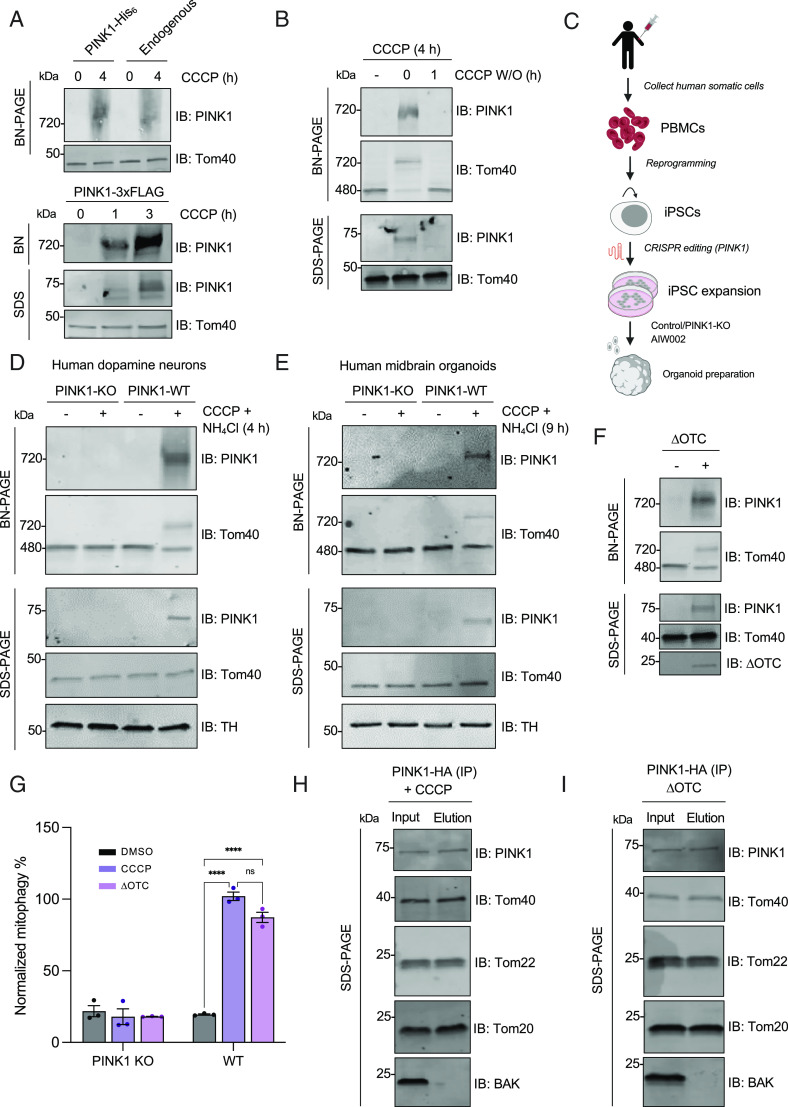
HMW PINK1 complex formation is ubiquitous across cell types and assembles in response to depolarization or misfolded protein accumulation. (*A*) HEK293T cells (endogenous or expressing PINK1-His_6_ or PINK1-3×FLAG) were treated with 20 µM CCCP or DMSO for the indicated time points, lysed in 1% digitonin solubilization buffer or sodium dodecyl sulfate (SDS) sample buffer, subjected to BN-PAGE or SDS-PAGE, and immunoblotted. (*B*) U2OS cells expressing endogenous PINK1 were treated with 20 µM CCCP or for 4 h before washing out CCCP for the indicated times. Cells were lysed and immunoblotted for PINK1 and Tom40 as in *A*. (*C*) Schematic of hMBO generation from peripheral blood mononuclear cells which have been reprogrammed into iPSCs and CRISPR edited for PINK1 deletion. (*D*) Six-week-old PINK1 KO DA neurons were treated with 20 µM CCCP or DMSO, subjected to BN-PAGE or SDS-PAGE, and then immunoblotted. (*E*) hMBOs (PINK1-KO or WT) that were 8.5 wk old were treated with 20 µM CCCP and 5 mM ammonium chloride to inhibit lysosomal degradation as indicated, subjected to BN-PAGE and SDS-PAGE, and were immunoblotted using PINK1 and Tom40 antibodies. (*F*) U2OS cells were transiently transfected with vector or ΔOTC constructs for 36 h and then fractionated. Mitochondrial-enriched fractions were solubilized, used for BN-PAGE (*Upper*) and SDS-PAGE (*Lower*), and immunoblotted. (*G*) Quantification of mitophagy using the mt-Keima reporter assay in PINK1-HA transfected U2OS PINK1 KO cells following DMSO (black) or CCCP (violet) treatment, or ΔOTC transfection (pink). Bars indicate the relative level of mitophagy, normalized to WT PINK1 treated with CCCP, plotted as mean (n = 3) ± SEM. Two-way ANOVA with Tukey’s post hoc tests (n = 3), *P < 0.05; **P < 0.01; ***P < 0.001; ****P < 0.0001; ns, not significant. (*H*) U2OS PINK1 KO cells were transfected PINK1-HA or mock vector and treated with 20 µM CCCP for 4 h. Mitochondria were isolated, and HA immunocapture was performed. Bound proteins were eluted with HA peptide, and fractions were subjected to SDS-PAGE or BN-PAGE immunoblotting. (*I*) Mock or PINK1-HA transfected U2OS PINK1 KO cells were transfected with ΔOTC for 36 h followed by mitochondrial isolation and immunocapture. Bound proteins were eluted and subjected to SDS-PAGE immunoblotting for the antibodies indicated.

To validate previous reports of HMW PINK1 complex assembly upon depolarization and to investigate the dynamics of PINK1 accumulation, we transfected full-length PINK1 with either a C-terminal 3× FLAG tag or 6×-HIS fusion tag in HEK293T cells, treated with 20 µM CCCP or DMSO and monitored HMW PINK1 complex formation at various time points by blue native-poly-acrylamide gel electrophoresis (BN-PAGE) (45). As predicted, both endogenous and the different tagged-PINK1 proteins assembled into a complex with an approximate molecular mass of 720 kDa (Fig. 1*A*). This complex likely contains the core TOM complex, as the endogenous Tom40 subunit can be observed to shift its migration pattern upon CCCP treatment from 450 kDa, the approximate mass of the native TOM complex, to comigrate with PINK1 at 720 kDa (Fig. 1*B*). Proteinase K treatment led to the degradation of the HMW PINK1 complex, but not the inner membrane protein Tim22 (*SI Appendix*, Fig. S1), indicating that the 720-kDa complex is exposed to the cytosolic side of the OMM in depolarized mitochondria. As reported previously (18), brief washout of CCCP to reestablish ΔΨm led to the disassembly of the endogenous HMW PINK1 complex, reaccumulation of the native 450-kDa TOM complex, and degradation of PINK1 (Fig. 1*B*).

To further characterize the endogenous HMW PINK1 complex in more physiological and PD-relevant cellular models, we used human iPSC-derived DA neurons and human midbrain organoids (hMBOs) (Fig. 1*C*) (46). As a control, we used an isogenically matched PINK1 KO line, generated in the same iPSC background (47). Using immunofluorescence, we found that more than 75% of the neurons from both wild-type (WT) and PINK1 KO iPSCs were tyrosine hydroxylase-positive, indicating that the loss of PINK1 did not affect neuronal differentiation into DA neurons (*SI Appendix*, Fig. S2). Both WT and PINK1 KO DA neurons and hMBOs were treated with either DMSO or CCCP and ammonium chloride to halt lysosomal degradation and increase PINK1 signal. The appearance of a CCCP/NH_4_Cl-dependent, PINK1- and Tom40-positive 720-kDa HMW complex could be observed on BN-PAGE in 6-wk-old WT DA neurons (Fig. 1*D*) and 8.5-wk-old hMBOs (Fig. 1*E*) but not in PINK1 KO DA neurons or hMBOs. Interestingly, the mobility of Tom40 itself did not shift in response to CCCP in the absence of PINK1. This suggests that despite the arrest of ΔΨm-dependent import of a myriad of proteins into mitochondria, no other endogenous TOM substrate, at least in neural human tissue, can substitute for the loss of PINK1 in the assembly of a stable complex with TOM. More broadly, these results in human DA neurons and hMBOs further reenforce the robustness of the observed HMW PINK1 complex across models, including those with high relevance to PD.

### Misfolded Mitochondrial Protein Accumulation Elicits PINK1-TOM Complex Formation.

Besides mitochondrial uncoupler-mediated import arrest, other stressors such as misfolded mitochondrial protein accumulation, have been reported to induce PINK1 stabilization on the OMM (48). Specifically, expression of a deletion mutant of ornithine transcarbamylase (ΔOTC), a protein that misfolds and accumulates in the mitochondrial matrix and induces the mitochondrial unfolded protein response (UPR^mt^) in mammalian cells, leads to PINK1 accumulation and Parkin recruitment on the OMM without disruption of membrane potential (48, 49). We hypothesized that ΔOTC expression would also result in the assembly of the HMW PINK1 complex. To address this, U2OS PINK1 KO cells expressing PINK1-HA were transiently transfected with a plasmid encoding ΔOTC, followed by mitochondrial extraction and BN-PAGE or SDS-PAGE. Similar to CCCP treatment, ΔOTC expression induced the formation of a 720-kDa HMW PINK1 complex (Fig. 1*F*). Moreover, both CCCP and ΔOTC elicited comparable levels of PINK1-dependent mitophagy (Fig. 1*G* and *SI Appendix*, Fig. S3*A*), as measured by flow-cytometry using the mt-Keima reporter in U2OS cells expressing exogenous green fluorescent protein (GFP)-Parkin (50). Using HA-immunopurification from mitochondrial lysates extracted from U2OS PINK1 KO cells expressing PINK1-HA, either treated with CCCP (Fig. 1*H*) or coexpressing ΔOTC (Fig. 1*I*), we found that both stressors induced similar coelution profiles and comparable enrichment patterns of TOM complex subunits (specifically Tom20, Tom40, and Tom22). However, in contrast to CCCP, ΔOTC did not affect ΔΨm (*SI Appendix*, Fig. S3*B*), indicating that the two mitochondrial stressors triggered mitophagy via distinct mechanisms. Although previous reports validated the localization of ΔOTC aggregates within the mitochondrial matrix (48), we sought to determine whether ΔOTC aggregates could be a stable component of the HMW PINK1 complex. To this end, we performed BN-PAGE on U2OS cells overexpressing ΔOTC and PINK1. In our assay, ΔOTC migrated at <242 kDa, suggesting that ΔOTC does not stably interact with the PINK1-TOM assembly, nor the core TOM complex (*SI Appendix*, Fig. S3*C*). Given that these ΔOTC aggregates are known to be in the mitochondrial matrix and do not disrupt ΔΨm, this suggests that ΔOTC induces PINK1 accumulation not by blocking the TOM complex, but instead by disrupting the matrix chaperone machinery and/or import motor. Taken together, the findings demonstrate that ΔOTC induction of the UPR^mt^ results in a HMW PINK1 complex that contains a similar assortment of TOM subunits and appears competent to trigger mitophagy without requiring mitochondrial depolarization induced by CCCP.

### PINK1 Forms a Supercomplex with TOM and TIM23 upon Mitochondrial Depolarization.

**Fig. 2.**
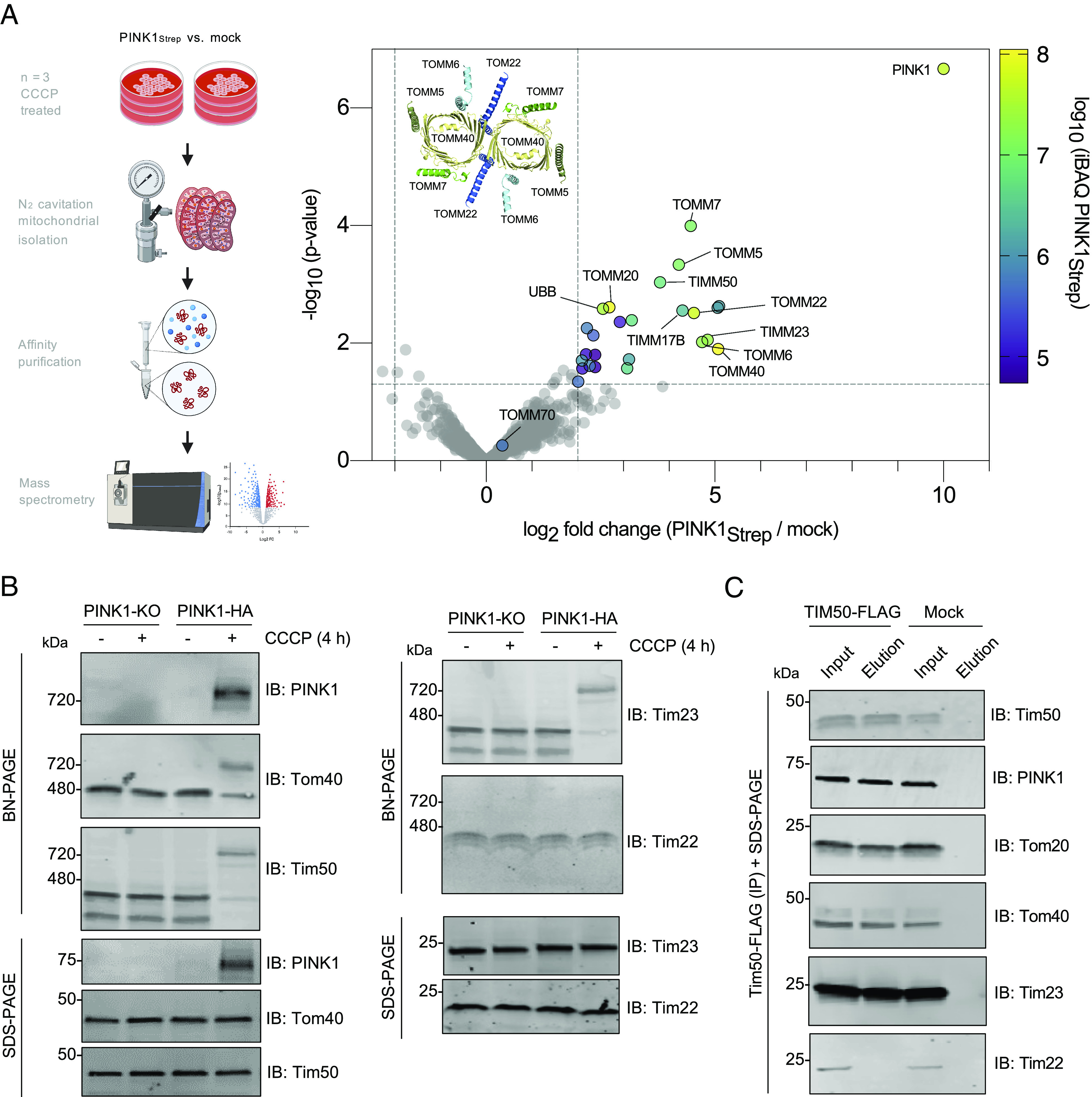
PINK1 forms a supercomplex with TOM and TIM23 complexes upon depolarization. (*A*) HEK293T cells were transfected with PINK1-Strep or mock vector, mitochondria were isolated using nitrogen cavitation, StrepTactin purification was performed, and eluates were subjected to mass spectrometry analysis (*Left*). LFQ was performed (n = 3) and results are plotted as a volcano plot. Significantly enriched hits (−log_10_ *P*-value > 1.3 and log_2_ fold change > 2, indicated by dashed lines) are colored according to their average intensity-based absolute quantification (iBAQ) intensity across PINK1-Strep samples and selected hits are labeled. The core human TOM complex (PDB: 7CK6) is depicted as cartoons and labeled for reference. (*B*) Mock or pCMV(d1) PINK1-HA transfected U2OS PINK1 KO cells were treated with 20 µM CCCP or DMSO as indicated, subjected to BN-PAGE or SDS-PAGE, and immunoblotted as indicated. (*C*) Mock or Tim50-FLAG transfected HEK293T cells were treated with 20 µM CCCP for 4 h followed by mitochondrial isolation and FLAG immunocapture. Bound proteins were eluted with FLAG peptide, and fractions were subjected to SDS-PAGE immunoblotting.

Previously, using a candidate approach, the HMW PINK1 complex was shown to contain components of the 450-kDa TOM complex (Tom40, Tom20, Tom22, Tom5, Tom6, Tom7, and Tom70) (18). Using an unbiased mass spectrometry–based approach, we sought to determine whether other components were part of the HMW PINK1 complex. Briefly, we isolated mitochondria from either mock or PINK1-2xStrep-His transfected, CCCP-treated HEK293T cells, affinity-purified PINK1, and subjected the elutions to LC–MS/MS analysis. Label-free quantification (LFQ) was performed to compare the enrichment in PINK1 versus mock-transfected cells (Fig. 2*A* and Dataset S1). First, our analysis reaffirmed the presence of previously known TOM subunits within the PINK1-TOM complex, as seen by the significant enrichment of Tom40, Tom22, Tom20, Tom5, Tom6, and Tom7 in the PINK1-transfected samples. Strikingly, three subunits of the TIM23 complex were also enriched within the CCCP-induced supercomplex, namely Tim50, Tim23, and Tim17B. This finding suggests that when PINK1 accumulates on mitochondria, it spans the entire mitochondrial IMS and tethers the TOM and TIM23 complexes as part of a PINK1-TOM-TIM23 supercomplex. Indeed, upon CCCP treatment, both endogenous Tim50 and Tim23 comigrated with PINK1 at 720 kDa on BN-PAGE consistent with the existence of a supercomplex containing PINK1 and components of TOM and TIM23 (Fig. 2*B*). In contrast, Tim22, a subunit of a distinct translocase complex (TIM22) at the IMM did not comigrate with PINK1, TOM or TIM23 subunits, indicating that supercomplex does not include TIM22. Next, we overexpressed Tim50-FLAG or Tim23-FLAG and performed a FLAG immunopurification in CCCP-treated cells to further validate the components of the supercomplex. As expected from the LC–MS/MS data, we copurified PINK1 along with Tom40 and Tom20 with both Tim50 (Fig. 2*C*) and Tim23 (*SI Appendix*, Fig. S4). Tim50 and Tim23 could reciprocally copurify each other but not the Tim22 subunit of the IMM TIM22 complex. While Tom70 may play a role in steady-state PINK1 import (51, 52), our LC–MS/MS data indicate that Tom70 is not part of the PINK1-TOM-TIM23 supercomplex, consistent with a previous antibody-based gel shift study (19). Similar to our results with the TOM complex, none of the examined TIM23 subunits’ mobility shifted in response to CCCP in the absence of PINK1 (Fig. 2*B*), again consistent with PINK1 functioning as an essential import substrate, required for the assembly of a HMW supercomplex in response to stress in mammalian cells.

### Tom20 Directly Interacts with PINK1 and Gates Downstream PINK1 Activity.

**Fig. 3.**
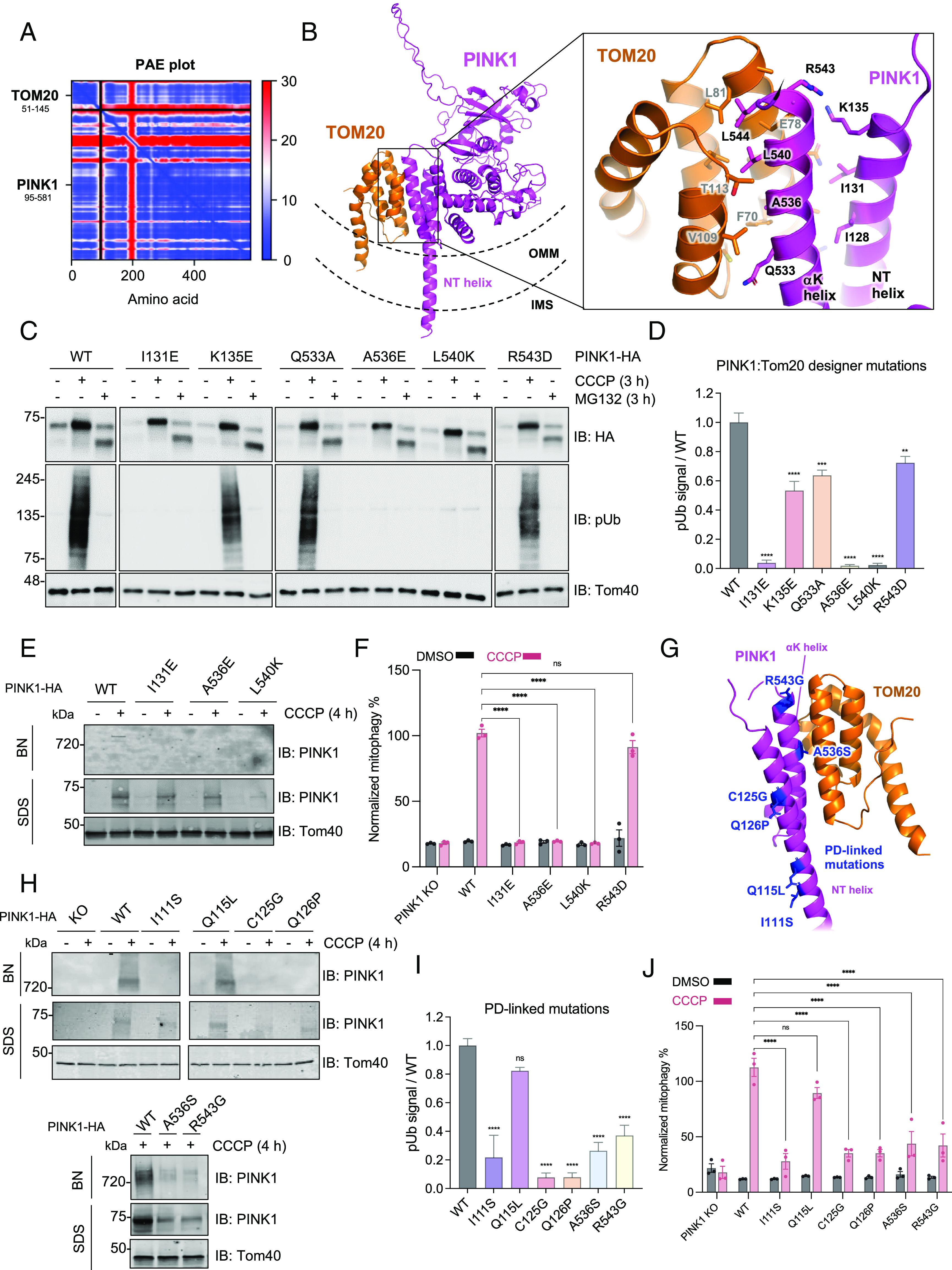
Characterization of the PINK1-Tom20 binding interface using designer and PD-linked mutations within the PINK1 NT-CTE. (*A*) AlphaFold multimer v3 was run on the cytosolic domains of PINK1 (a.a. 95 to 581) and Tom20 (a.a. 51 to 145) with 20 recycles and an RMSD tolerance between cycles of 0.5 Å. The top-ranked model was chosen for PAE plot visualization. (*B*) Structural visualization of the PINK1-Tom20 AlphaFold model using PyMOL. PINK1 and Tom20 are depicted in the context of the OMM and IMS topology. (*C*) U2OS PINK1 KO cells were transfected with pCMV(d1) PINK1-HA (WT or indicated mutants) and were treated with 20 μM CCCP or 10 µM MG132 for 4 h. Lysates were run on SDS-PAGE and immunoblotted as indicated. (*D*) Quantification of immunoblots by densitometry for *C*. Band intensities were normalized relative to the level of Tom40 in the CCCP-treated sample, and the ratio to pSer65 of WT CCCP-treated sample was calculated. Bars represent mean ± SEM (n = 3). One-way ANOVA with Dunnett’s post hoc test was performed (*P < 0.05; **P < 0.01; ***P < 0.001; ****P < 0.0001; ns, not significant). (*E*) U2OS PINK1 KO cells were transfected with pCMV(d1) PINK1-HA WT or mutants and treated with 20 μM CCCP for 4 h. Mitochondria were extracted, solubilized, and analyzed via BN-PAGE or SDS-PAGE immunoblotting. (*F*) U2OS cells transfected with pCMV(d1) PINK1 (WT or mutants) were treated with 20 μM CCCP or DMSO for 4 h and mitophagy was quantified using the mt-Keima reporter assays. Bars indicate the relative level of mitophagy, normalized to WT PINK1 treated with CCCP, plotted as mean (n = 3) ± SEM. Two-way ANOVA with Tukey’s post hoc tests (n = 3), *P < 0.05; **P < 0.01; ***P < 0.001; ****P < 0.0001; ns, not significant. (*G*) PD-linked mutations visualized within the PINK1 NT-CTE and TOM20 AlphaFold model. (*H*) U2OS PINK1 KO cells were transfected with pCMV(d1) PINK1-HA (WT or indicated mutants) and treated with 20 μM CCCP for 4 h. Mitochondria were extracted, solubilized, and analyzed by immunoblotting. (*I*) U2OS PINK1 KO cells were transfected with pCMV(d1) PINK1-HA (WT or indicated mutants) and treated with 20 μM CCCP for 4 h. Lysates were immunoblotted and quantified as in *D*. (*J*) U2OS PINK1 KO cells were transfected with pCMV(d1) PINK1 mutants and assayed using the mt-Keima reporter assay and quantified as in *F*.

To provide structural context for our mass spectrometry data, we ran AlphaFold Multimer predictions iteratively using PINK1 as a bait against the top 30 of our significantly enriched mass spectrometry hits (*SI Appendix*, Fig. S5). Strikingly, the highest scoring predictions from our unbiased screen were for complexes of PINK1-Ub and PINK1-Tom20. As the Ub binding site on insect PINK1 was previously determined (28), we decided to focus on the PINK1-Tom20 interaction, as it was the only TOM subunit previously shown to cross-link to PINK1 and form a direct interaction as part of the HMW PINK1 complex (18). Our AlphaFold prediction for PINK1 (95 to 581) and the cytosolic domain of Tom20 (51 to 145) also produced a high-confidence model, with all top five ranks converging on the same solution, as visualized by the low error scores in the predicted aligned error (PAE) plot (Fig. 3*A*). In this model, PINK1 binds to the Tom20 presequence binding groove via its NT helix (a.a. 118 to 135) and αK helix (a.a. 524 to 544 in the CTE) (Fig. 3*B*). It is known that Tom20 is a labile component of the TOM complex with high lateral mobility, suggesting that it could dissociate and reassociate with the core TOM complex depending on the presence of presequences at the TOM gate (53). This model is also consistent with the recent cryogenic electron microscopy (cryo-EM) structures of human TOM in which Tom20 copurified with the TOM complex without chemical cross-linking, but only generated resolvable electron density upon cross-linking to Tom40 and Tom22 (54, 55). In our model, PINK1 accumulation could stabilize the interaction of Tom20 with the rest of the core TOM complex. Conversely, we hypothesized that the binding of PINK1 to Tom20 upon depolarization and the formation of a stable PINK1-Tom20 complex at the OMM would stabilize PINK1 folding on the OMM, effectively gating downstream PINK1 activity and mitophagy.

To test this experimentally, we introduced a series of charged residues in place of key NT and αK residues that were predicted to interact with Tom20 in our AlphaFold model (Fig. 3*B*). We then transfected these mutants into U2OS PINK1 KO cells and quantified the levels of Phospho-S65 Ub (pUb) as a readout of PINK1 activity. Three mutations (I131E, A536E, and L540K) were completely deficient in pUb generation despite the CCCP-dependent accumulation of full-length PINK1 on SDS-PAGE (Fig. 3 *C* and *D*). These three same mutants also failed to support the assembly of the PINK1-TOM-TIM23 supercomplex on BN-PAGE (Fig. 3*E*) and were deficient in downstream mitophagy, as measured via the mt-Keima reporter assay in U2OS cells expressing exogenous GFP-Parkin (Fig. 3*F*). These mutants still localized to mitochondria following subcellular fractionation (*SI Appendix*, Fig. S6*A*) and were capable of being cleaved by PARL as indicated by the formation of the 52-kDa PINK1 fragment upon MG132 treatment (Fig. 3*C*). Furthermore, these mutants remained accessible to proteinase K digestion in isolated mitochondria, implying that these mutants are still found on the external surface of the OMM (*SI Appendix*, Fig. S6*B*). We did notice a reduction in the amounts of accumulated PINK1 L540K following mitochondrial isolation, both in our proteinase K protection and BN-PAGE assays (Fig. 3*E* and *SI Appendix*, Fig. S6 *A* and *B*) even though the levels were unchanged in whole cell lysates (Fig. 3*D*). Yet, the reduction in PINK1 L540K levels is not sufficient to explain the strong reduction in pUb generation and mitophagy. Overall, these results show that the PINK1 NT-CTE:Tom20 interface is critical for the formation of the PINK1-TOM-TIM23 supercomplex and kinase activation for downstream mitophagy.

In order to substantiate the relevance of this PINK1-Tom20 module in the context of PD, we also tested six PD-linked mutations (56) or naturally occurring variants (Fig. 3*G*) within the NT and αK helices for their effects on PINK1-TOM-TIM supercomplex assembly, PINK1 pUb generation, and downstream mitophagy. I111S, C125G, and Q126P are located in the NT helix and are likely pathogenic for PD (57–59). Q115L is also in the NT helix but is equally frequent in PD patients and controls, suggesting that it is benign (60). Finally, the ClinVar database lists two variants of uncertain significance located in the CTE, A536S, and R543G, which could impact binding to Tom20 (61). Five (I111S, C125G, Q126P, A536S, and R543G) of the six mutants were deficient in forming the PINK1-TOM-TIM23 supercomplex (Fig. 3*H*). Moreover, these same five PD mutants were deficient in pUb generation (Fig. 3*I*) and in downstream mitophagy (Fig. 3*J*). We confirmed that I111S, C125G, and Q126P exhibited a severe defect in Parkin recruitment to mitochondria (*SI Appendix*, Fig. S7) and a reduced colocalization with mitochondria on confocal immunofluorescence microscopy (*SI Appendix*, Fig. S8). Conversely, the benign variant Q115L could support Ub phosphorylation, PINK1-TOM-TIM23 supercomplex assembly, and mitophagy and only showed a slight delay in Parkin recruitment (*SI Appendix*, Fig. S7). Based on the structural model of PINK1-Tom20 (Fig. 3*G*), neither I111S nor Q115L would affect Tom20 binding, but I111S is located further upstream and could disrupt an interaction with other TOM subunits. As shown in previous reports (32), introducing a proline into the NT helix (Q126P) prevented PINK1 from accumulating on SDS-PAGE (*SI Appendix*, Fig. S6*C*), suggesting that loss of NT helicity could unfold PINK1 entirely, or at least disrupt both the NT-Tom20 interaction and the 3E motif in the NT helix which stalls PINK1 within the Tom40 pore. Taken together, these findings underscore Tom20 as a crucial interactor of PINK1, acting as a ratchet that helps stabilize PINK1 on the TOM complex when import via TIM23 is stalled long enough to allow folding of the kinase domain via the NT-CTE module. This folding and stabilization of PINK1, in turn, would enable autophosphorylation, Ub phosphorylation, and downstream mitophagy. These findings are consistent with previous results regarding the regulation of mitophagy through celastrol-mediated modulation of PINK1-TOM20 binding (62).

### PINK1 Is Required to Tether the TOM and TIM23 Complexes.

**Fig. 4.**
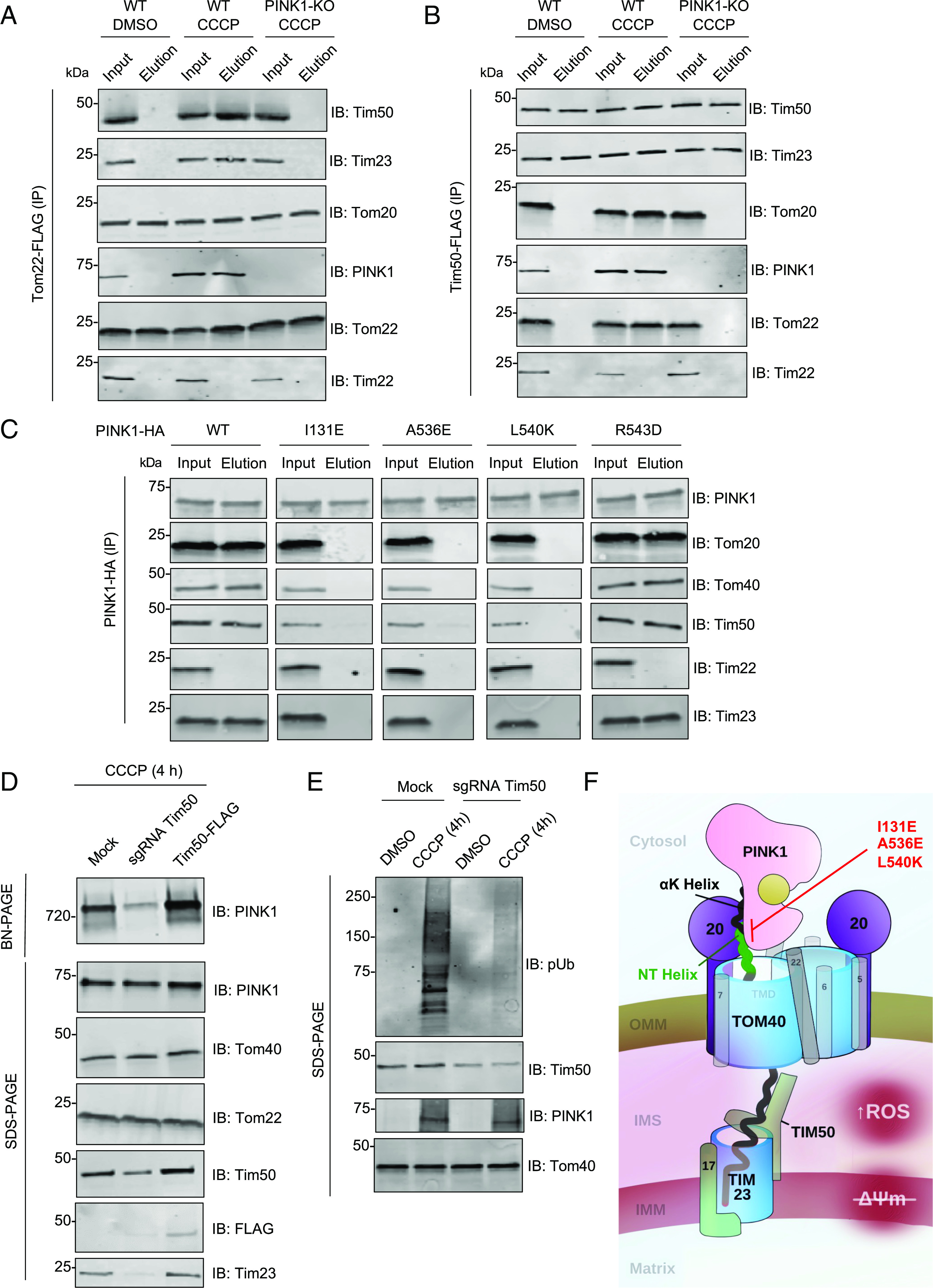
PINK1 endogenously tethers TOM and TIM23 following import arrest in a Tom20-dependent manner. (*A*) U2OS cells (WT or PINK1-KO) were transfected with Tom22-FLAG and treated with 20 µM CCCP or DMSO for 4 h, followed by mitochondrial isolation and FLAG immunocapture. Bound proteins were eluted with FLAG peptides and subjected to SDS-PAGE immunoblotting. (*B*) U2OS cells (WT or PINK1-KO) were transfected with Tim50-FLAG and treated with 20 µM CCCP or DMSO for 4 h, followed by mitochondrial isolation and FLAG immunocapture as in Fig. 3*A*. (*C*) U2OS PINK1 KO cells transfected with pCMV(d1) PINK1-HA (WT or Tom20 binding deficient mutants) were treated with 20 µM CCCP for 4 h followed by mitochondrial isolation and HA immunocapture. Bound proteins were eluted with HA peptides and subjected to SDS-PAGE immunoblotting. (*D*) U2OS PINK1 KO cells expressing PINK1-HA were transfected with mock vector, CRISPRi sgRNA targeting Tim50, or Tim50-Flag, and treated with 20 µM CCCP or DMSO for 4 h. Lysates were subjected to SDS-PAGE or BN-PAGE immunoblotting as previously described. (*E*) U2OS PINK1 KO cells expressing PINK1-HA were transfected with mock vector or CRISPRi sgRNA targeting Tim50 and treated with 20 µM CCCP or DMSO for 4 h. Lysates were subjected to SDS-PAGE or BN-PAGE immunoblotting for the antibodies indicated as previously described. (*F*) Simplified schematic of PINK1 topology within the PINK1-TOM-TIM supercomplex. Accessory TOM subunits are depicted as gray. PINK1 NT and αK helices are labeled along with Tom20 (depicted as “20”). This model of Tom20 binding places the TMD of PINK1 within the Tom40 pore in the OMM.

The translocation of protein precursors into mitochondria requires the transfer of precursors from the TOM translocase at the OMM to the translocation machineries at the IMM, including the TIM23 complex, the main IMM translocase for presequence-containing precursors (63). Despite their localization on different mitochondrial membranes, under certain circumstances, usually in the context of overexpression of artificial or synthetic stalled import substrates in yeast, TOM and TIM23 have been shown to assemble into a supercomplex, tethered by the artificial substrate trapped in their import channels (64–66). Given our observation that the assembly of TOM and TIM23 into a stable supercomplex in response to mitochondrial stress requires PINK1 (Figs. 1 *D* and *E* and 2*B*), we asked whether PINK1 could serve as a bona fide native tethering import substrate in mammalian cells. To address this, we performed Tom22-FLAG and Tim50-FLAG immunopurifications under DMSO and CCCP conditions in both WT and PINK1 KO U2OS cells. As expected, Tom22 copurified with Tom20, another subunit of the TOM complex, both at baseline and after CCCP treatment (Fig. 4*A*). In contrast, Tom22 only copurified PINK1 and the TIM23 subunits, Tim23 and Tim50, upon depolarization with CCCP, confirming the requirement of mitochondrial stress for PINK1-TOM-TIM23 supercomplex assembly (Fig. 4*A*). Reciprocally, Tim50 copurified the TIM23 subunit, Tim23, at baseline but only copurified PINK1 and the TOM subunits, Tom20 and Tom22, upon depolarization (Fig. 4*B*). Under none of the conditions did Tom22 or Tim50 copurify the Tim22 subunit of the TIM22 translocase at IMM. Most importantly, in the absence of PINK1, neither Tom22 nor Tim50 copurified subunits from the opposing complex, strongly arguing that PINK1 is required to tether the TOM and TIM23 complexes in response to mitochondrial stress (Fig. 4 *A* and *B*). Next, we determined whether the Tom20-PINK1 NT-CTE interaction described above (Fig. 3) was critical for PINK1 to tether the TOM and TIM23 complexes by performing PINK1-HA immunopurifications in CCCP-treated U2OS PINK1 KO cells transfected with either WT or the I131E, A536E, L540K, and R543D PINK1 mutants. As expected, Tom20 copurification was abrogated in the three mutants (I131E, A536E, and L540K) which showed deficient pUb generation, PINK1-TOM-TIM23 supercomplex formation, and mitophagy (Fig. 3 *C–F*), but not in R543D, a mutant whose function was similar to WT PINK1 (Fig. 4*C*). Furthermore, the defective PINK1 mutants also prevented the copurification of Tom40, suggesting that the Tom20-PINK1 NT-CTE interaction is required for PINK1 to stably bind the TOM complex. Finally, even if these same three mutants were properly localized to mitochondria (Fig. 3*C*), they failed the copurify the Tim50 and Tim23 subunits of the TIM23 complex, suggesting that Tom20-PINK1 NT-CTE binding is required for PINK1 to tether TOM and TIM23 into a supercomplex. We also performed a pulldown of these Tom20-binding deficient PINK1 mutants using Tim50-FLAG immunopurification (*SI Appendix*, Fig. S9). In the presence of these PINK1 mutants, Tim50-FLAG copurified with Tim23 but not Tom40 or PINK1, confirming that stable TOM-TIM23 supercomplex formation hinges on the PINK1-Tom20 interaction. Next, we sought to characterize how modulation of TIM23 complex levels would impact the PINK1-dependent tethering of TOM-TIM23. To this end, we knocked down Tim50 using CRISPRi or overexpressed Tim50-FLAG in CCCP-treated cells and visualized PINK1-TOM-TIM23 formation by BN-PAGE. Whereas Tim50 knockdown did not reduce PINK1 levels overall, as seen on SDS-PAGE, it did reduce the amount of PINK1-TOM-TIM23 supercomplex on BN-PAGE (Fig. 4*D*) and the amount of pUb generated (Fig. 4*E*). Furthermore, overexpressing Tim50-FLAG lead to an apparent increase in PINK1-TOM-TIM23 supercomplex levels (Fig. 4*D*). It is critical to note that Tim50 knockdown also reduced the levels of the Tim23 subunit, which makes it difficult to draw conclusions on whether this reduction is Tim50 specific or simply due to disruption of the stoichiometry of the TIM23 complex. Nonetheless, these findings suggest that the entire TIM23 complex is one of the key factors for the stabilization of PINK1 on the TOM complex. In line with this, another recent study revealed that reduction of Tim23 levels also attenuated PINK1-TOM-TIM23 complex formation, though the effects of Tim50 knockdown on supercomplex assembly were not characterized (39). It was also proposed that PINK1 which escaped Tim23 binding was susceptible to OMA1 proteolysis, which suggests that the abundance of the TIM23 complex and its ability to bind PINK1 are critical for maintaining the stability of accumulated PINK1 within the supercomplex. Future studies will be essential to clarify the specific roles of Tim23 and Tim50 in PINK1 accumulation and PINK1-TOM-TIM23 assembly.

### Concluding Remarks.

Taken together, our findings establish that in response to mitochondrial stress, PINK1 accumulates and tethers core components of the TOM and TIM23 complexes, leading to the assembly of a 720-kDa PINK1-TOM-TIM23 supercomplex (Fig. 4*E*). The complex forms across physiologically relevant cell models, including DA neurons and hMBOs and different stressors, including depolarization and the ΔOTC-induced UPR^mt^. These findings reinforce the broad significance of this biochemical assembly in neuronal mitochondria and the conjecture that the PINK1-TOM-TIM23 complex serves as an integrator of multiple mitochondrial stress signals. It will be crucial to further characterize the potential differences in subunit composition between different cell types and between CCCP- and ΔOTC-induced PINK1 assemblies at the level of TIMs and the matrix import motor.

While TOM-TIM23 supercomplexes have been visualized previously in yeast using electron cryotomography (67) or cross-linking to a synthetic import intermediate (Jac1 fused to superfolding GFP) (64), PINK1 represents an endogenous physiological import substrate to tether and stabilize TOM-TIM23 supercomplex assembly with high occupancy following mitochondrial stress. Although the precise oligomeric states of the TOM and TIM23 machinery in complex with PINK1 remain to be determined, based on the apparent 720-kDa molecular mass for PINK1-TOM-TIM23 on BN-PAGE and the list of cointeractors from our mass spectrometry data, it is likely that the TOM machinery is present as a tetramer in its PINK1-bound state, as TOM dimers or trimers containing PINK1 and TIM23/17/50 heterotrimers should migrate at <550 kDa in molecular mass. This would be consistent with the observation of TOM tetramers by cryoelectron microscopy, in both yeast and human purified TOM complexes (55, 68). To this end, deciphering the precise stoichiometry of the HMW PINK1 complex will be crucial to understand the physiological ramifications of these mitochondrial assemblies.

TOM and TIM23 tethering by PINK1 clearly depends on the Tom20-PINK1 NT-CTE interaction on the OMM side of the supercomplex and possibly on Tim50 on the IMS/IMM side. While Tom20 is known to bind presequences to facilitate protein import, our work positions PINK1 as a noncanonical import substrate that bridges TOM and TIM23 complexes upon mitochondrial stress in a Tom20-dependent manner. Thus, we envision that in healthy mitochondria, competent for import, PINK1 would be efficiently pulled through the TOM complex via AFG3L2 or other TIM machinery before the NT-CTE had sufficient time to fold completely, effectively dislodging the Tom20 interaction to expose PINK1 to the PARL active site in the IMM for subsequent PINK1 cleavage and degradation. In contrast, in mitochondria where import is compromised, the fully folded PINK1 NT-CTE would remain bound to Tom20 in an orientation where the PINK1 TMD is not accessible for cleavage, effectively allowing PINK1 to remain Tom20-bound, its kinase domain to fold properly at the cytosolic side of the OMM and trigger downstream mitophagy. This model sets the stage for further studies regarding the mechanisms by which the mitochondrial import machinery senses misfolded protein-induced mitochondrial damage signals. Future studies will also be warranted to study the functional impact of PINK1 PD mutations on the PINK1 complex formation in DA neurons and its subsequent impact on neuronal mitochondrial biology, a process which will likely provide key functional insights into the molecular basis of PD.

Overall, our work yields key insights into the determinants for PINK1-TOM-TIM23 supercomplex assembly and downstream mitophagy as well as shedding light on yet another aspect of how mitochondrial translocases fine-tune PINK1-mediated mitophagy, laying the foundation for future studies on mitochondrial quality control in PD biology.

## Materials and Methods

### Cell Culture and Immunoblotting.

U2OS PINK1 KO cell lines were generated by CRISPR as previously reported (37). Cells were cultured in DMEM (Dulbecco’s Modified Eagle Medium) supplemented with 10% fetal bovine serum and 1× Pen/Strep and grown at 37 °C and 5% CO_2_. pCMV(d1)TNT PINK1(WT)-3HA, denoted as PINK1-HA throughout the manuscript, was obtained from Noriyuki Matsuda for attenuated PINK1 expression (69). PCR mutagenesis was used to generate mutations in the NT-CTE of human PINK1 in the pCMV(d1)TNT PINK1 vector. Other plasmids used include pReceiver M14 PINK1-3×FLAG (GeneCopoeia), denoted as PINK1-3×FLAG, and pTT5-PINK1-2×Strep-His, denoted as PINK1-Strep or PINK1-HIS. For expression of a misfolded protein model, the deletion mutant pOTCΔ_(30–114)_ (ΔOTC) and the WT OTC were obtained from Addgene (49). U2OS PINK1 KO cells were transfected with 1 µg pCMV(d1)TNT PINK1(WT)-3HA or indicated plasmids in six-well plates for 48 h, followed by treatment with 20 µM of CCCP or equal volume of DMSO for 4 h. Whole cells were harvested, resolved by SDS-PAGE and immunoblotted on nitrocellulose as described previously (70). The antibodies indicated below were used: PINK1 D8G3 (Cell Signaling cat# 6946), Anti-Tom40 Antibody (H-7): sc-365466; Phospho-Ub Ser65 (E2J6T) (Cell Signaling, cat# 62802), Anti-Tom70 Antibody (A-8): sc-390545; Anti-Tom22 Antibody (Sigma, cat# T6319); Anti-Tom20 antibody (Santa Cruz Biotechnology, sc-17764), Anti-HA 6E2 (Cell Signaling cat# 2367), Anti-Tim23 (Abcam cat# ab230253), Anti-Tim22 (Abcam, cat# ab251909) and Anti-Tim50 (Santa Cruz Biotechnology, sc-393678), Anti-BAK antibody (Cell Signaling, cat #3814), and Anti-OTC (Sigma, HPA000243). The membranes were blocked with 3% fish skin gelatin (Sigma) in 1× phosphate buffered saline (PBS) with 0.1% Triton X-100), probed with primary and secondary antibodies, and imaged with an Odyssey infrared imaging system using the manufacturer’s recommended procedures (LI-COR). For Fig. 3*C* and all phospho-S65 Ub (pUb) blots, proteins were resolved on 4 to 20% (for pUb) or 10% SDS-PAGE (for TOM40 and HA) gels, transferred for 90 min at 250 mA onto polyvinylidene difluoride (PVDF) membranes, blocked in 1× TBS-T (0.1% Tween 20) with 5% bovine serum albumin (BSA), and incubated with primary antibodies overnight at 4 °C. The following day, membranes were washed, incubated with HRP-conjugated secondary antibody for 1 h at room temperature, washed again, and then visualized using ClarityTM chemiluminescence (Bio-Rad).

### Mitochondrial Isolation and BN-PAGE.

U2OS PINK1 KO cells were transfected with pCMV(d1)TNT PINK1(WT)-3HA or the indicated mutants for 36 h, followed by treatment with 20 µM of CCCP or equal volume of dimethyl sulfoxide (DMSO) for 4 h. The cells were then harvested in mitochondrial isolation buffer containing 20 mM 4-(2-hydroxyethyl)-1-piperazineethanesulfonic acid (HEPES) (pH 7.4), 220 mM mannitol, 70 mM sucrose, and cOmplete protease inhibitor cocktail ethylenediaminetetraacetic acid (EDTA)-free (Roche). Nitrogen cavitation was performed to pellet mitochondria as described previously (37, 71). The mitochondria were then solubilized in a buffer containing 20 mM BIS-TRIS (pH 7.3), 100 mM NaCl, 10% glycerol, protease inhibitors, and 1% digitonin and left on a rotor for 3 h at 4 °C. The suspension was spun at 5,000 g for 5 min at 4 °C and the supernatants were collected. BN-PAGE was performed using Native™ PAGE Running Buffer (Invitrogen) containing 0.002% G-250 (Invitrogen). Gels were shaken in denaturation buffer (10 mm Tris-HCl pH 6.8, 0.1% SDS, and 0.006% 2-mercaptoethanol) for 60 min after electrophoresis and then transferred to PVDF membranes for immunoblotting. The membranes were blocked with 3% fish skin gelatin (Sigma) in 1× PBS with 0.1% Triton X-100, probed with primary and secondary antibodies, and imaged with an Odyssey infrared imaging system (LI-COR).

### hMBO, Mitochondrial Extraction, and BN-PAGE.

Detailed procedures regarding hMBO, mitochondrial extraction, and BN-PAGE are indicated in **SI Appendix*, Materials and Methods*.

### Dopaminergic Neuron Generation from Induced Pluripotent Stem Cells.

The previously validated AIW002 control and PINK1-KO pluripotent stem cell (iPSC) lines were used (47). IPSC culture and neuronal differentiation were conducted according to previously established protocols (72–74). Further detailed procedures are indicated in **SI Appendix*, Materials and Methods*.

### Mitophagy Assay Using the mt-Keima Reporter.

Mitophagy was examined using a FACS-based analysis of mitochondrially targeted Keima (mt-Keima) as previously described in refs. 50, 75, and 76. Briefly, U2OS PINK1 KO cells stably expressing GFP-Parkin (WT) and ecdysone-inducible mt-Keima were transfected with PINK1-HA (WT or indicated mutants) for 36 h, induced with 10 μM ponasterone A, and treated with 20 μM CCCP for 4 h. For FACS analysis, cells were trypsinized, washed, and resuspended in PBS prior to their analysis on a LSR Fortessa (BD Bioscience) equipped with 405 and 561 nm lasers and 610/20 filters. Measurement of lysosomal mitochondrially targeted mt-Keima was made using a dual excitation ratiometric pH measurement where pH 7 was detected through the excitation at 405 nm and pH 4 at 561 nm. For each sample, 10,000 events were collected and single, GFP-Parkin-positive cells were subsequently gated for mt-Keima. Data were analyzed using FlowJo v10.1 (Tree Star). Data were collected in triplicate and each individual value was normalized to the mean of WT PINK1 + CCCP from Fig. 3*F*. For statistical analysis, two-way ANOVA with Tukey’s post hoc tests were performed.

### Immunoprecipitation Experiments.

For FLAG immunopurification experiments, mock transfected HEK293T cells and PINK1-3×FLAG expressing cells were treated with 20 µM CCCP for 4 h followed by mitochondrial isolation and immunocapture using Anti-FLAG M2 Affinity gel. Bound proteins were eluted with FLAG peptides-containing elution buffer (containing 0.2% digitonin), and various fractions as indicated were subjected to BN-PAGE or SDS-PAGE followed by immunoblotting using PINK1, Tom22, Tom40, and FLAG antibodies.

For HA-immunopurification experiments, mock transfected U2OS PINK1 KO and PINK1-HA transfected cells were treated with 20 µM CCCP for 4 h followed by mitochondrial isolation and immunocapture using Pierce™ Anti-HA Magnetic Beads. Bound proteins were eluted with Pierce HA peptides (final concentration 2 mg/mL)-containing elution buffer (containing 0.2% digitonin), and various fractions as indicated were subjected to BN-PAGE or SDS-PAGE followed by immunoblotting using PINK1, Tom22, Tom40, and Bak antibodies.

For His-immunopurification experiments, mock transfected HEK293T cells and PINK1-HIS transfected HEK293T cells were treated with 20 µM CCCP for 4 h followed by mitochondrial isolation and immunocapture using His CoPur affinity resin. Bound proteins were eluted with HIS-Select® Elution Buffer (containing 0.2% digitonin), and various fractions as indicated were subjected to BN-PAGE or SDS-PAGE followed by immunoblotting using PINK1, Tom22, and Tom40 antibodies.

### HMW PINK1 Complex Purification for Mass Spectrometry.

HEK293T cells were mock transfected or transfected in triplicate with 30 µg of pTT5-PINK1-2×Strep-His (denoted as PINK1-Strep) using Lipofectamine 3000 according to the manufacturer’s instructions. After 36 h, cells were treated with 20 µM CCCP for 3 h and then harvested and subjected to mitochondrial isolation by nitrogen cavitation (as described above). Mitochondrial pellets were solubilized in lysis buffer with 1% digitonin [25 mM HEPES pH 7.5, 150 mM NaCl, 5% glycerol, 1 mM tris (2-carboxyethyl)phosphine (TCEP), supplemented with PhosStop (Roche), cOmplete protease inhibitor cocktail EDTA-free (Roche), and 1 mM EDTA]. Lysates were incubated for 90 min with end-over-end mixing and were spun at 15,000 rpm for 20 min. Supernatants were diluted to 0.5% digitonin and were loaded onto Strep-Tactin resin (IBA). The resin was washed three times with 25 mM HEPES pH 7.5, 150 mM NaCl, 5% glycerol, 1 mM TCEP, 1 mM EDTA, and 0.1% digitonin. Samples were eluted in wash buffer containing 10 mM desthiobiotin. Eluted fractions were pooled, and proteins were precipitated using methanol-chloroform extraction. Pellets were resolubilized in 8 M urea + 0.1% ProteaseMAX surfactant (Promega) and were reduced with dithiothreitol (DTT) and alkylated with iodoacetamide according to the manufacturer’s instructions. 200 ng of Trypsin/Lys-C mix (Promega) was added, and the digestion was incubated at 37 °C overnight. Trifluoroacetic acid was added to a final concentration of 0.5% to stop the digestion and samples were dried in a LabConco Centrivap DNA vacuum concentrator. Dried pellets were resuspended in 0.5% formic acid/5% acetonitrile and were cleaned using C18 spin columns (Pierce), according to the manufacturer’s instructions. Eluates were again dried in a LabConco Centrivap DNA vacuum concentrator.

### Mass Spectrometry Analysis.

Extracted peptides were resolubilized in 0.1% aqueous formic acid/2% acetonitrile and loaded onto a Thermo Acclaim Pepmap (Thermo, 75 μm × 2 cm C18 3 μm beads) precolumn and then onto an Acclaim Pepmap EASY-Spray (Thermo, 75 μm × 15 cm with 2 μm C18 beads) analytical column separation using a Dionex Ultimate 3000 uHPLC at 250 nL/min with a gradient of 2 to 35% organic (0.1% formic acid in acetonitrile) over 1 h. Peptides were analyzed using a Thermo Orbitrap Fusion mass spectrometer operating at 120,000 resolutions (full width at half maximum (FWHM) in MS1) with higher-energy collisional dissociation (HCD) sequencing (15,000 resolution) at top speed for all peptides with a charge of 2+ or greater.

The raw data were processed using Andromeda, integrated into MaxQuant (version 2.1.4.0). Searches for tryptic (before K, R) peptides were performed against the H. sapiens proteome, with a minimum peptide length of 7 a.a. and a maximum of two missed cleavages. Cysteine carbamidomethylation was selected as a fixed modification. Protein N-term acetylation, methionine oxidation, phosphorylation (STY), and deamidation (NQ) were chosen as variable modifications. Default Orbitrap instrument parameters were used, including a first search peptide mass tolerance of 20 ppm and main search peptide tolerance of 4.5 ppm. Peptide spectral match and protein false discovery rate thresholds were set to 1%. LFQ was performed using only unique peptides for quantification. Match Between Runs was also used with default values.

MaxQuant LFQ values were analyzed using LFQ-Analyst as previously described (77). To summarize, data were prefiltered to remove contaminants, reverse sequences, proteins only “identified by site,” proteins quantified with only a single peptide, and proteins with a high proportion of missing values. All LFQ values were transformed to a log 2 scale, and missing values were imputed using the “Missing Not at Random” method, frequently used in Perseus software (78). Protein-wise linear models combined with empirical Bayes statistics were used for the differential expression analyses. Volcano plots were generated in GraphPad Prism using *P*-values and fold enrichments from LFQ-Analyst and iBAQ values from MaxQuant. The mass spectrometry proteomics data have been deposited to the ProteomeXchange Consortium via the PRIDE (79) partner repository with the dataset identifier PXD047765.

### Tim50 CRISPRi Cloning and Transfection.

Detailed procedures are indicated in *SI Appendix*, *Materials and Methods*.

## Supplementary Material

Appendix 01 (PDF)

Dataset S01 (XLSX)

## Data Availability

All study data are included in the article and/or supporting information. The mass spectrometry proteomics data have been deposited to the ProteomeXchange Consortium via the PRIDE partner repository (79) with the dataset identifier PXD047765.
