# Supplementary material for: Tom20 gates PINK1 activity and mediates its tethering of the TOM and TIM23 translocases upon mitochondrial stress
Source: Proc Natl Acad Sci U S A. 2024 Feb 28;121(10):e2313540121. doi: 10.1073/pnas.2313540121 (PMC10927582; doi:10.1073/pnas.2313540121)
Supplement: Supplementary file 1 — Appendix 01 (PDF) [file pnas.2313540121.sapp.pdf]

SI Appendix file

SI Materials and Methods:

Human midbrain organoid generation, mitochondrial extraction and blue-native PAGE.

Human midbrain organoids (hMBOs) were derived from the healthy control cell line AIW002-02 reprogrammed from human peripheral blood monocytes into iPSCs (1) and an isogenic CRISPR-induced deletion of PINK1 in the AIW002-02 line (2). hHMBOs were generated from iPSC cells using Enuvio embryoid-body disks (EB-DIST) on day 0 and transferred to bioreactors after 8 days following the previously reported protocol (3). After 8.5-weeks, organoids were transferred to media containing 20 µM CCCP and 5 mM NH_4Cl or equivalent DMSO for 9 h. After treatment, organoids were washed multiple times with PBS and then harvested in mitochondrial isolation buffer containing 20 mM HEPES (pH 7.4), 220 mM mannitol, 70 mM sucrose and cOmplete protease inhibitor cocktail EDTA-free (Roche). Nitrogen cavitation was performed on ice for 5 minutes at a pressure of 500 psi. The resulting suspension was centrifuged for 5 minutes at 3000 rpm and the pellet was resuspended in mitochondrial isolation buffer. Mitochondrial fractions were pelleted by centrifugation for at 12000 rpm for 20 minutes. The mitochondria were then solubilized in a buffer containing 20 mM BIS-TRIS (pH 7.3), 100 mM NaCl, 10% glycerol, protease inhibitors and 1% digitonin and left on a rotor for 3 hours at 4°C. The suspension was spun down at 5000 g for 5 minutes at 4°C and the supernatants were carried forward for BN-PAGE gels.

Mitochondrial GFP-Parkin recruitment time-lapse microscopy.

U2OS PINK1 KO cells were seeded on a 35-mm Glass Bottom 4 Compartment Dish (Greiner Bio-One). Cells were co-transfected with WT eGFP-Parkin and pCMV(d1)TNT PINK1(WT)-3HA or mutants. 48 h after transfection, cells were stained with MitoTracker DeepRed FM (ThermoFisher) at a final concentration of 50 nM following manufacturer's guidelines. Cells were then transferred to a heated stage maintained at 37 °C and 5% CO_2 using a Zeiss temperature controller and cell perfusion system. Cells were treated with CCCP at a final concentration of 15 uM. Microscopy was performed on a Zeiss AxioObserver.Z1 inverted fluorescent microscope. Fully automated multidimensional acquisition was controlled using the Zen Pro software (Zeiss). Fixed exposure times were as follows: GFP 150 ms; and MitoTracker 40 ms. Images were taken at 4 min intervals for a total of 120 min. Parkin recruitment was visualized by the appearance of punctate GFP fluorescence overlapping Mitotracker fluorescence. The percentage of cells exhibiting Parkin recruitment to the mitochondria was calculated at 4 min intervals over 120 mins. Approximately 200 cells were examined per mutant over four separate and biologically independent experiments. The fluorescence intensity of GFP-positive puncta was not considered in Parkin recruitment analysis. For statistical analysis, a two-way analysis of variance (ANOVA) with Bonferroni post-test was performed, *$P<0.05$.

Dopaminergic neuron generation from induced pluripotent stem cells.

The previously validated AIW002 control and PINK1-KO pluripotent stem cell (iPSC) lines were used (2). IPSC culture and neuronal differentiation were conducted according to previously established protocols (4–6). Briefly, iPSCs were passaged using Relsr, plated on Matrigel coated dishes and maintained in mTeSR basal media. Midbrain neuronal precursor cells (NPCs) were generated following a previously established protocol. Briefly, iPSCs were grown until 70% confluent and then dissociated using gentle cell dissociation reagent and transferred to uncoated flasks in NPC Induction Media (Composed of DMEM/F12 supplemented with 1x N2, 1x B-27, 1x MEM NEAA solution, 200 ng/mL noggin, 200 ng/mL SHH, 3 µM CHIR-99021, 10 µM SB431542 and 100 ng/mL FGF-8) containing 10 µM of Y-27632 (ROCK inhibitor) to allow for embryoid body (EB) formation. Media was refreshed every 48 h and on the 7th day, EBs were transferred to polyornithine/laminin coated flask, allowing EBs to attach to the culture surface. Media was again changed every 48 h and on the 7th day EBs were dissociated into small colonies by trituration in gentle cell dissociation media, and replated on polyornithine/laminin coated flasks, splitting 1 flask of EBs into 2-3 flasks for expansion. The resulting NPC monolayer was grown again for 7 days in NPC induction media until 100% confluent, at which time NPCs were harvested with gentle cell dissociation media and frozen in FBS with 10 % DMSO.

For differentiation into dopaminergic neurons, NPCs were thawed in NPC maintenance media (composed of DMEM/F12 supplemented with 1x N2, 1x B-27, 1x MEM NEAA solution, 100 ng/mL FGF-8 and 2 µM purmorphamine), plated on polyornithine/laminin coated flasks and expanded for at least 7 days with media change every 24-48 h as needed. To plate NPCs for final differentiation into dopaminergic neurons, NPCs were dissociated using Accutase, counted and plated on polyornithine/laminin coated dishes at the desired density in dopaminergic differentiation media (Composed of Neurobasal A media supplemented with 1x B27, 1x N2, 1x Antibiotic-Antimycotic, 20 ng/mL BDNF, 20 ng/mL GDNF, 200 µM Ascorbic acid, 0.5 mM db-cAMP and 0.1 µM Compound E). After 5 days, media was supplemented with 0.1 µg/mL mitomycin C to remove any remaining proliferative cells. Dopaminergic neurons were maintained and matured by refreshing 1/3rd of the culture media every 5-7 days.

For imaging experiments neurons were plated on 96-well plates at a density of 15,000 cells per well. For biochemical experiments neurons were plated on either 6-well plates at a density of 750,000 cells per well or 15 cm dishes at a density of 10 million cells per plate, and then were treated with 20 µM CCCP and 5 mM NH_4Cl or DMSO for 4 h, harvested, lysed, and subjected to SDS-PAGE or BN-PAGE analysis in the same manner as U2OS PINK1 KO cells described above.

Characterization of neurons by immunofluorescence.

Neuronal identity was assessed by immunofluorescent staining for Map2 (neurons) and tyrosine hydroxylase (TH - dopaminergic neurons). After 4 weeks of differentiation neurons were fixed with 4% paraformaldehyde for 20 mins, permeabilized for 10 mins with 0.3% saponin in PBS, and blocked for 1 h with 1% BSA and 4% goat-serum in PBS. Primary antibodies against TH

(Pelfreeze, P40101) and Map2 (Encor, CPCA-MAP2) were diluted (1:500 and 1:2000 respectively) in blocking solution and incubated at 4 degrees overnight. Neurons were then washed 3 times in PBS and incubated for 1 hour with AlexaFluor secondary antibodies in blocking solution. Neurons were then washed twice and stained with Hoechst before imaging.

Imaging was performed on an Opera Phenix high content confocal microscope using 20X water immersion objective. Image analysis was performed using the Columbus software and data processing was then conducted using R studio. Briefly, nuclei were first identified by the Hoechst channel, and surrounding somal area was identified by Map2 staining. TH labelling intensity was then quantified within this Map2-defined region. Single-cell data were then exported as text files and imported into R studio for processing. A pre-processing script was used to filter objects based on nuclear size, nuclear shape and Map2 staining intensity to identify neuronal cells, and TH positivity was used to define the percentage of dopaminergic neurons.

Confocal imaging experiments and analysis.

For imaging experiments, U2OS PINK1 KO cells seeded at a density of 40,000 cells per 18mm glass coverslip in a 12 well plate were transiently transfected with 0.5 ug of plasmid DNA expressing WT-HA PINK1 under the control of a CMV weakened promoter using FuGene HD (Promega, E2311) at a ratio of 1:3. The media was changed after 2 h of transfection, and 20uM CCCP treatment was performed 48 h post-transfection for a period of 3 h in the absence of serum. The coverslips were fixed and stained for endogenous Tom20 (Santa Cruz Biotechnology, sc-17764) and exogenous HA (Biolegend, 902301), detected with donkey anti-mouse Alexa 488 (Thermo Fisher Scientific, A21202) and donkey anti-rabbit Alexa 555 (Thermo Fisher Scientific, A31572) secondary antibodies. Nuclei were counterstained with Hoechst (Thermo Fisher Scientific, H3570). Fluorescence images were processed using FIJI (ImageJ, NIH). The Mander's Coefficients were obtained using JACoP. The mean difference in the Mander's Coefficient between CCCP-treated and untreated cells was plotted in R. Changes relative to the WT were compared via general linear hypotheses tests as previously (7).

AlphaFold multimer predictions on mass spectrometry hits.

Iterative AlphaFold predictions for PINK1 against significantly enriched mass spectrometry hits were run on a local implementation of ColabFold Multimer using the AlphaFold framework (8, 9). Briefly, the full length PINK1 sequence was run against the sequences of the top 30 most enriched proteins with p-values < 0.05. Multiple sequence alignments were generated using mmseqs and used as input for the structure search using "colabfold_batch". Parameters were set to 20 recycles with an RMSD tolerance of 0.5 Å, as previously described (10). PINK1:prey protein complex predictions were ranked by their average multimer score across the top 3 generated models and were visualized as a heat map in GraphPad Prism.

Tim50 CRISPRi cloning and transfection.

The dCas9 plasmid used was pLX_311-KRAB-dCas9 (Addgene #96918, gift from John Doench & William Hahn & David Root; referred to as CRISPRi). gRNA plasmid cloning was adapted from Weissman lab protocols, using the pCRISPRi/a-v2 plasmid (Addgene #84832, gift from Jonathan Weissman). Two sgRNA's were used to target TIMM50, sgRNA1 (5'-GTCCGGGACGCCTCACCTCA-3'), and sgRNA2 (5'-GTGGCGTCAGCGCAAGATGG-3'). For transfection of CRISPRi machinery and sgRNA plasmids, ~5x10^6 HEK293T cells were seeded in a 150 mm dish, and after 24 h were co-transfected with dCas9, sgRNA1 and sgRNA2 in equimolar amounts using jetPRIME transfection reagent (Polyplus). Transfection was performed according to manufacturer's instructions, and cells were harvested 36 h post-transfection for following mitochondrial isolation and SDS-PAGE and BN-PAGE immunoblotting.

References

1. C. X.-Q. Chen, *et al.*, A Multistep Workflow to Evaluate Newly Generated iPSCs and Their Ability to Generate Different Cell Types. *Methods Protoc.* **4**, 50 (2021).

2. C. X.-Q. Chen, *et al.*, Generation of homozygous PRKN, PINK1 and double PINK1/PRKN knockout cell lines from healthy induced pluripotent stem cells using CRISPR/Cas9 editing. *Stem Cell Res.* **62**, 102806 (2022).

3. N.-V. Mohamed, *et al.*, Microfabricated disk technology: Rapid scale up in midbrain organoid generation. *Methods San Diego Calif* **203**, 465–477 (2022).

4. X. Chen, C. Rocha, T. Rao, T. M. Durcan, NeuroEDDU protocols_iPSC culture (2019) https:/doi.org/10.5281/zenodo.3738269 (July 14, 2023).

5. X. Chen, *et al.*, Induction of Dopaminergic or Cortical neuronal progenitors from iPSCs (2019) https:/doi.org/10.5281/zenodo.3738358 (July 14, 2023).

6. X. Chen, N. Lauinger, C. Rocha, T. Rao, T. M. Durcan, Generation of dopaminergic or cortical neurons from neuronal progenitors (2019) https:/doi.org/10.5281/zenodo.3738323 (July 14, 2023).

7. T. Hothorn, F. Bretz, P. Westfall, Simultaneous inference in general parametric models. *Biom. J. Biom. Z.* **50**, 346–363 (2008).

8. M. Mirdita, *et al.*, ColabFold: making protein folding accessible to all. *Nat. Methods* **19**, 679–682 (2022).

9. J. Jumper, *et al.*, Highly accurate protein structure prediction with AlphaFold. *Nature* **596**, 583–589 (2021).

10. R. Yin, B. Y. Feng, A. Varshney, B. G. Pierce, Benchmarking AlphaFold for protein complex modeling reveals accuracy determinants. *Protein Sci.* **31**, e4379 (2022).

SI Figure legends:

Supplemental Figure 1. Mitochondria were isolated from U2OS WT cells that were either treated with CCCP or vehicle control (DMSO). Isolated mitochondria were treated with or without external protease (PK, 20mg/ml) solubilized in 1% digitonin containing buffer and subjected to BN-PAGE and immunoblotting using antibodies against PINK1 (outer membrane), and TIM22 complex (inner membrane).

Supplemental Figure 2. Neuronal identity was assessed by immunofluorescent staining for Map2 (neurons) and tyrosine hydroxylase (TH - dopaminergic neurons). After 4 weeks of differentiation neurons were fixed with 4% paraformaldehyde for 20 mins, permeabilized for 10 mins with 0.3% saponin in PBS, and blocked for 1 h with 1% BSA and 4% goat-serum in PBS. Primary antibodies against TH (Pelfreeze, P40101) and Map2 (Encor, CPCA-MAP2) were diluted (1:500 and 1:2000 respectively) in blocking solution and incubated at 4 degrees overnight. Neurons were then washed 3 times in PBS and incubated for 1 hour with AlexaFluor secondary antibodies in blocking solution. Neurons were then washed twice and stained with Hoechst before imaging. Imaging was performed on an Opera Phenix high content confocal microscope using 20X water immersion objective. Image analysis was performed using the Columbus software and data processing was then conducted using R studio.

Supplemental Figure 3. A) U2OS cells transfected with pCMV(d1) PINK1 (WT or the indicated constructs), WT full-length OTC (FL-OTC) or ΔOTC were treated with 20 μM CCCP or DMSO for 4 h and were subjected to mt-Keima reporter assays. Bars indicate the relative level of mitophagy, normalized to WT PINK1 treated with CCCP, plotted as mean (n = 2). **B)** ΔOTC, full length OTC (FL-OTC) expressing cells and cells treated with 20 μM CCCP (4 h) cells were detached, stained with TMRE, and analyzed for the intensity of TMRE using a fluorescence plate reader as recommended by the manufacturer. As a negative control, untreated cells were stained with TMRE before FACS analysis. FACS results were represented as mean ± SEM from three independent experiments. **C)** U2OS PINK1 KO cells expressing PINK1-HA were either mock transfected or transfected with ΔOTC for 36 h. Mitochondria were isolated with nitrogen cavitation, and samples were subjected to BN-PAGE and SDS-PAGE immunoblotting. Antibody used for detecting ΔOTC was: Anti-OTC (Sigma, HPA000243).

Supplemental Figure 4. Mock or Tim23-FLAG transfected HEK293T cells were treated with 20 μM CCCP for 4 h followed by mitochondrial isolation and immunocapture using M2 FLAG affinity gel. Bound proteins were eluted with FLAG peptide and various fractions were subjected to SDS-PAGE immunoblotting using the indicated antibodies.

Supplemental Figure 5. ColabFold multimer was run locally using full-length PINK1 as a bait against all enriched mass spectrometry hits. Complexes were sorted according to the average of their Multimer scores across the top 3 models. PINK1:Tom20 and PINK1:Ub structures were visualized in PyMOL.

Supplemental Figure 6. A) U2OS PINK1 KO cells were transfected for 24 hours with designated PINK1-HA mutants and treated with 20 µM CCCP for 3 h, as previously described. Cytosolic and mitochondrial fractions following nitrogen cavitation were collected, normalized using the BCA assay, and subjected to immunoblotting. Antibodies used in this assay were: Anti-HA (6E2, Cell Signalling #2367); Anti-Tom20 (D8T4N, Cell Signalling #42406); STAT3 (9d8, Abcam Ab119352). **B)** Isolated CCCP-treated mitochondria expressing PINK1-HA mutants were pelleted and re-suspended in mitochondrial isolation buffer with or without 100 µg/mL of proteinase K (NEB). Samples were incubated on ice for 30 min, and then proteinase K digestion was stopped by addition of 5 mM PMSF. Mitochondria were pelleted again, re-suspended in 1X Laemmli SDS sample buffer, boiled at 95 °C for 5 min, and subjected to immunoblotting. Antibodies used in this assay were: Anti-HA (6E2, Cell Signalling #2367); Anti-Tom20 (D8T4N, Cell Signalling #42406); Anti-COXIV (3E11, Cell Signalling #4850). **C)** U2OS PINK1 KO cells were transfected with designated PINK1-HA mutants, treated with DMSO, 20 µM CCCP, or 10 µM MG132 for 4 h and subjected to immunoblotting as previously described.

Supplemental Figure 7. Time-lapse imaging of Parkin recruitment to mitochondria upon treatment with 20 µM CCCP in U2OS PINK1 KO cells co-expressing with WT eGFP-Parkin and α-NTE PINK1 mutants (I111S, Q115L, C125G, Q126P) as indicated. Recruitment can be visualized by the appearance of punctate GFP fluorescence. Scale bar: 100 µm.

Supplemental Figure 8. Confocal microscopy of PINK KO U2OS cells that were either untransfected or transiently transfected with the indicated PINK1-HA constructs with or without CCCP treatment. The scale bar represents 5 µm. B. The bars indicate the mean differences in the Mander's Coefficient between CCCP-treated and untreated cells, and the standard error of the mean differences. Changes relative to the WT were compared via general linear hypotheses tests (*** all adjusted $p < 1e\text{-}7$; $t > 5.82$).

Supplemental Figure 9. U2OS PINK1 KO cells were co-transfected with indicated PINK1 mutants and Tim50-FLAG for 48 h, treated with 20 µM CCCP for 4 h and mitochondria were isolated by N_2 cavitation. Mitochondrial pellets were lysed in 1 % digitonin, and lysates were subjected to immunocapture using Anti-FLAG M2 Affinity gel. Bound proteins were eluted with FLAG peptides-containing elution buffer (containing 0.2% digitonin) and various fractions as indicated were subjected to SDS-PAGE followed by immunoblotting using the indicated antibodies.

Figure S1

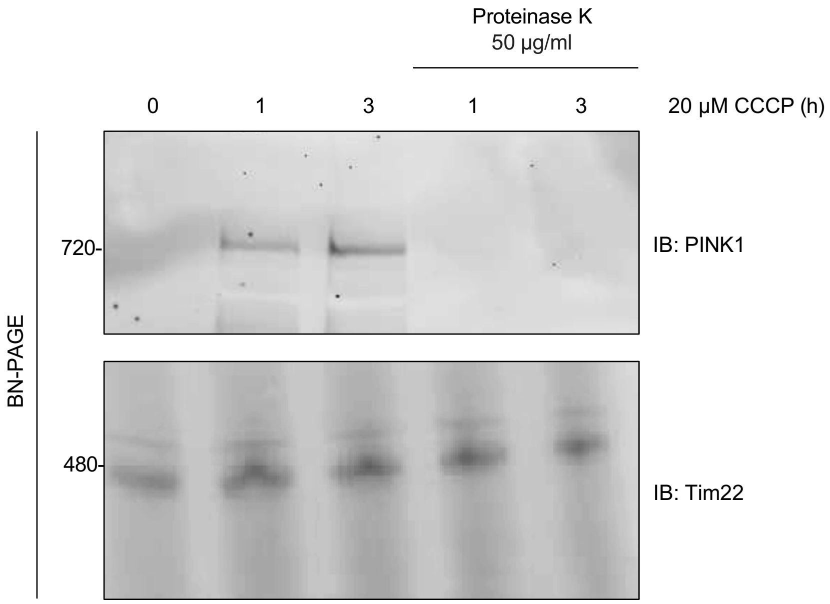
Proteinase K
50 µg/ml
0
1
3
1
3
20 µM CCCP (h)
BN-PAGE
720-
IB: PINK1
480-
IB: Tim22

Figure S2

A

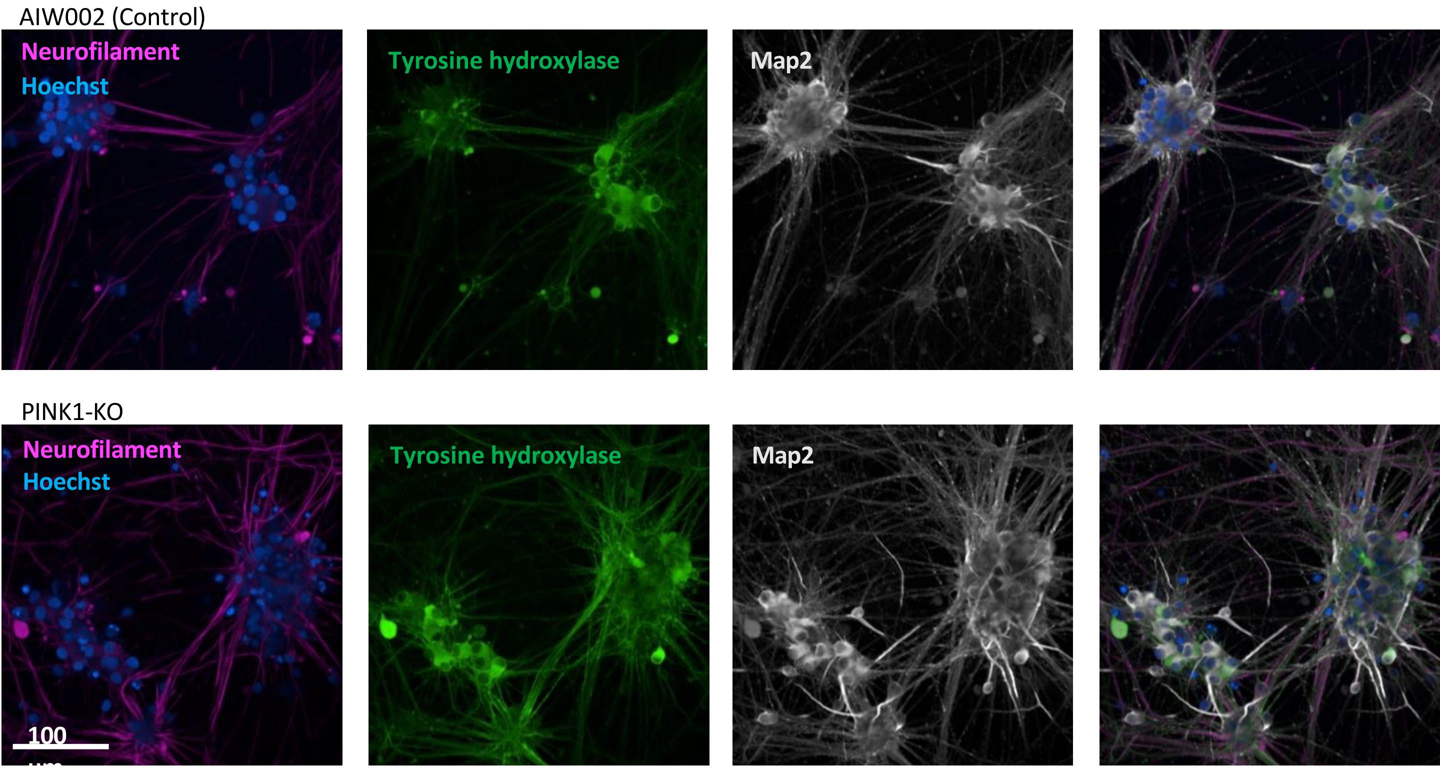
AIW002 (Control)
Neurofilament
Hoechst
Tyrosine hydroxylase
Map2
PINK1-KO
Neurofilament
Hoechst
Tyrosine hydroxylase
Map2
100

B

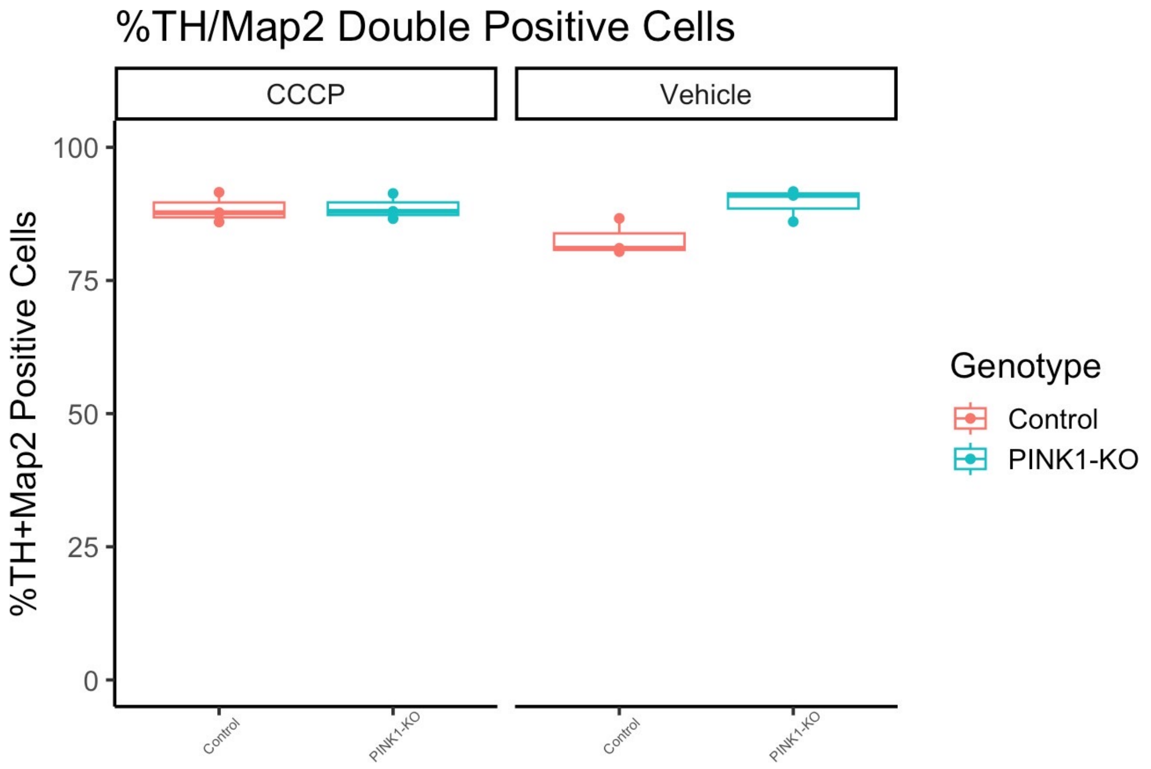
%TH/Map2 Double Positive Cells
CCCP
Vehicle
%TH+Map2 Positive Cells
100
75
50
25
0
Control
PINK1-KO
Control
PINK1-KO
Genotype
Control
PINK1-KO

Figure S3

A

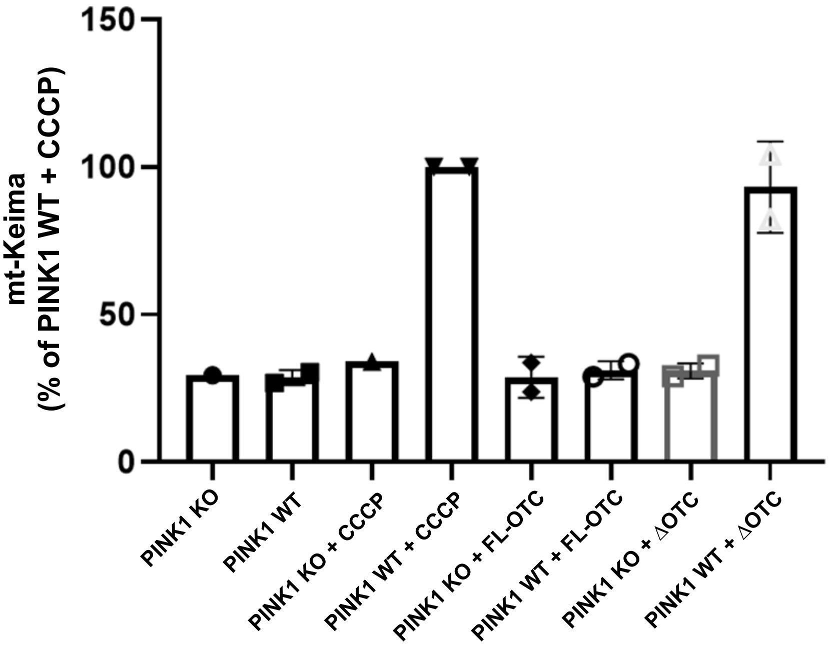

B

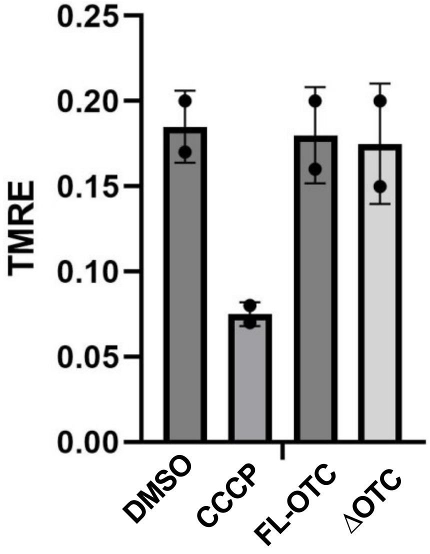

C

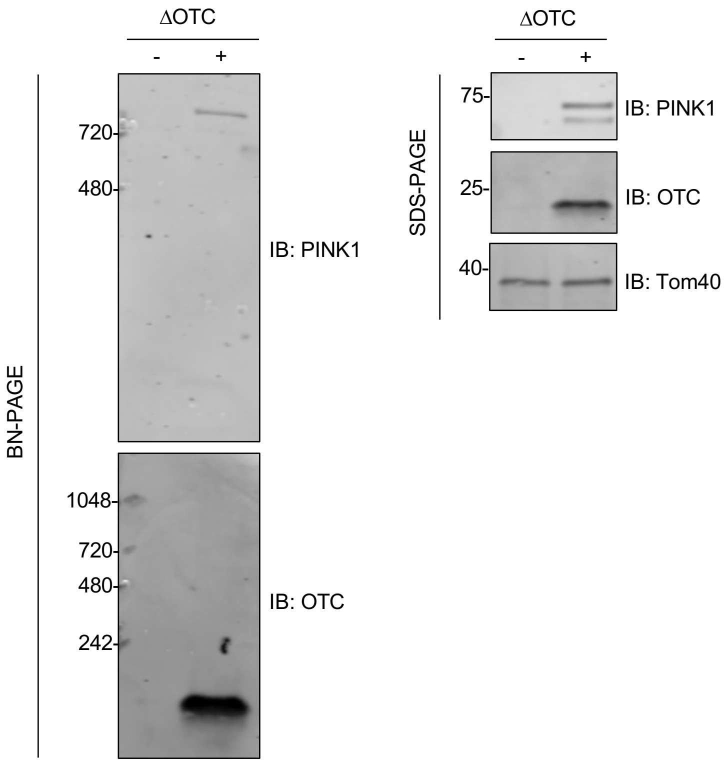

Figure S4

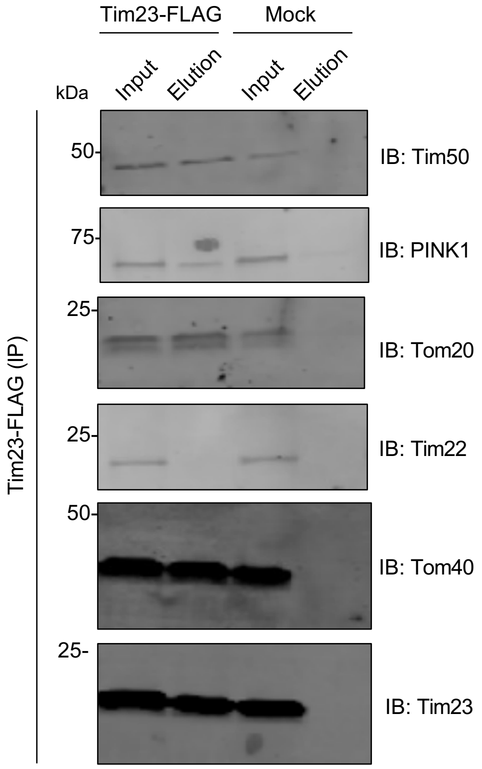

Tim23-FLAG
Mock
kDa
Input
Elution
Input
Elution
50-
IB: Tim50
75-
IB: PINK1
25-
IB: Tom20
25-
IB: Tim22
50-
IB: Tom40
25-
IB: Tim23
Tim23-FLAG (IP)

Figure S5

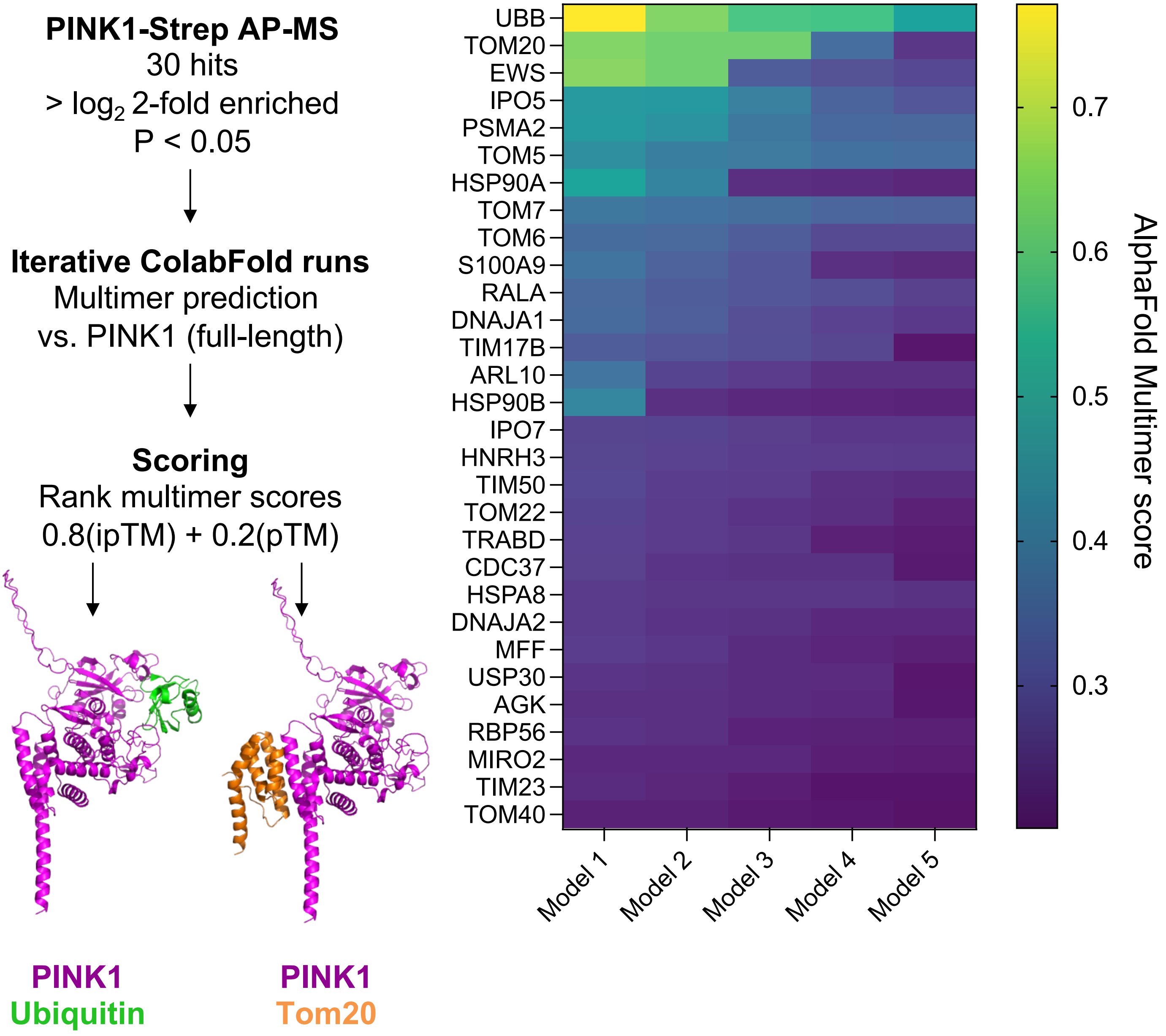

PINK1-Strep AP-MS
30 hits
> log2 2-fold enriched
P < 0.05
Iterative ColabFold runs
Multimer prediction
vs. PINK1 (full-length)
Scoring
Rank multimer scores
0.8(ipTM) + 0.2(pTM)
PINK1
Ubiquitin
PINK1
Tom20
UBB
TOM20
EWS
IPO5
PSMA2
TOM5
HSP90A
TOM7
TOM6
S100A9
RALA
DNAJA1
TIM17B
ARL10
HSP90B
IPO7
HNRH3
TIM50
TOM22
TRABD
CDC37
HSPA8
DNAJA2
MFF
USP30
AGK
RBP56
MIRO2
TIM23
TOM40
Model 1
Model 2
Model 3
Model 4
Model 5
0.7
0.6
0.5
0.4
0.3
AlphaFold Multimer score

Figure S6

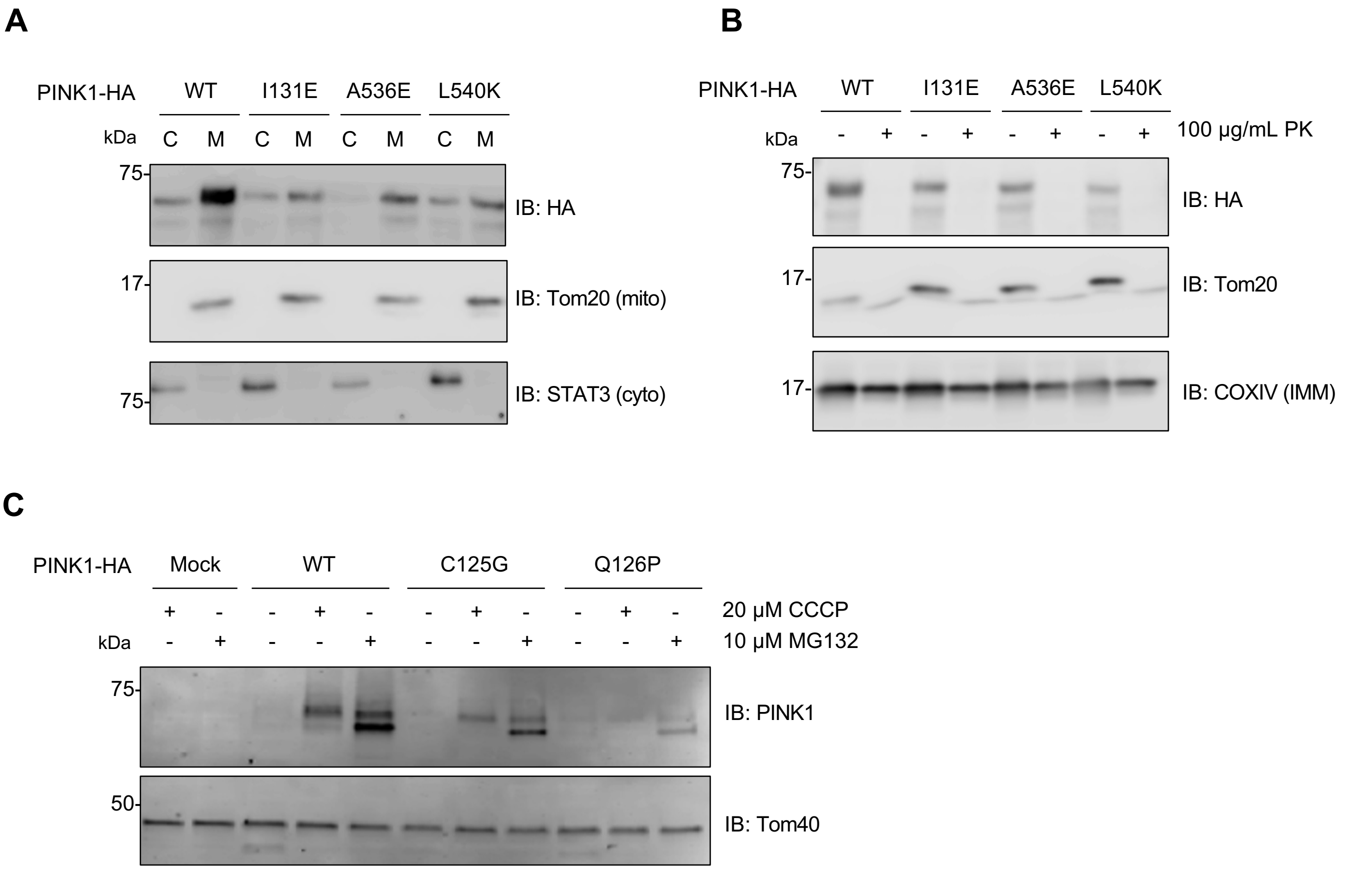
A
PINK1-HA
WT
I131E
A536E
L540K
kDa
C
M
C
M
C
M
C
M
75-
IB: HA
17-
IB: Tom20 (mito)
75-
IB: STAT3 (cyto)
B
PINK1-HA
WT
I131E
A536E
L540K
kDa
- + - + - + - +
100 µg/mL PK
75-
IB: HA
17-
IB: Tom20
17-
IB: COXIV (IMM)
C
PINK1-HA
Mock
WT
C125G
Q126P
+ - - + - - + - - + -
20 µM CCCP
kDa
- + - - + - - + - - +
10 µM MG132
75-
IB: PINK1
50-
IB: Tom40

Figure S7

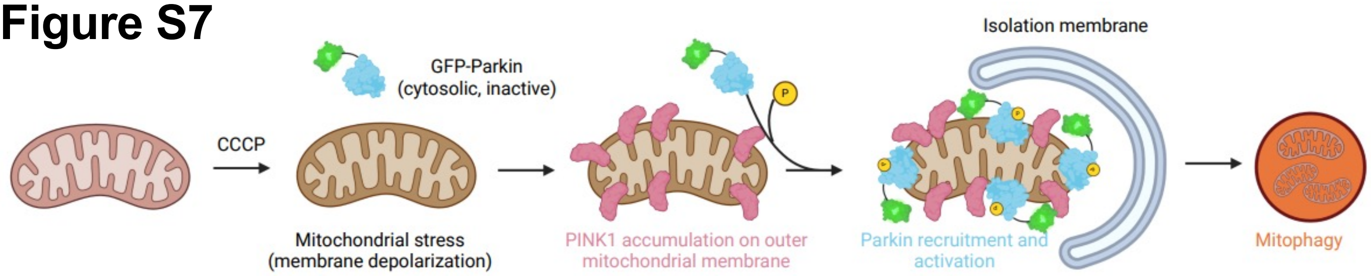
CCCP
GFP-Parkin
(cytosolic, inactive)
Mitochondrial stress
(membrane depolarization)
PINK1 accumulation on outer
mitochondrial membrane
Isolation membrane
Parkin recruitment and
activation
Mitophagy

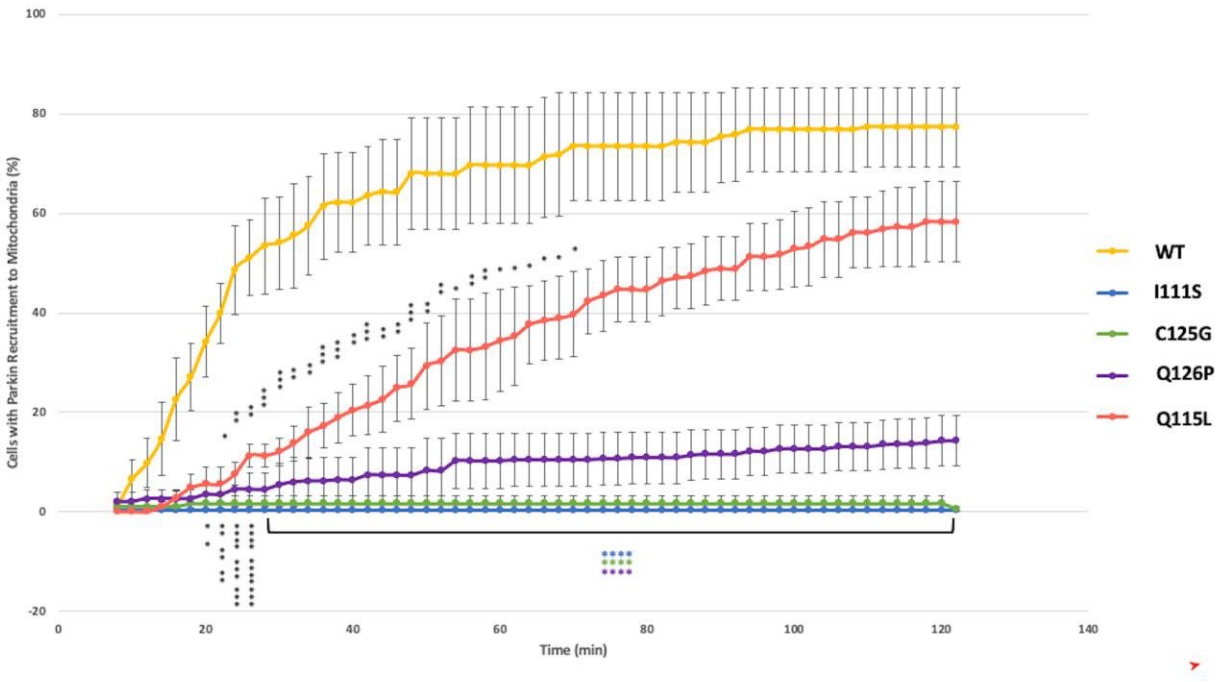
100
80
60
40
20
0
-20
Cells with Parkin Recruitment to Mitochondria (%)
0
20
40
60
80
100
120
140
Time (min)
WT
I111S
C125G
Q126P
Q115L

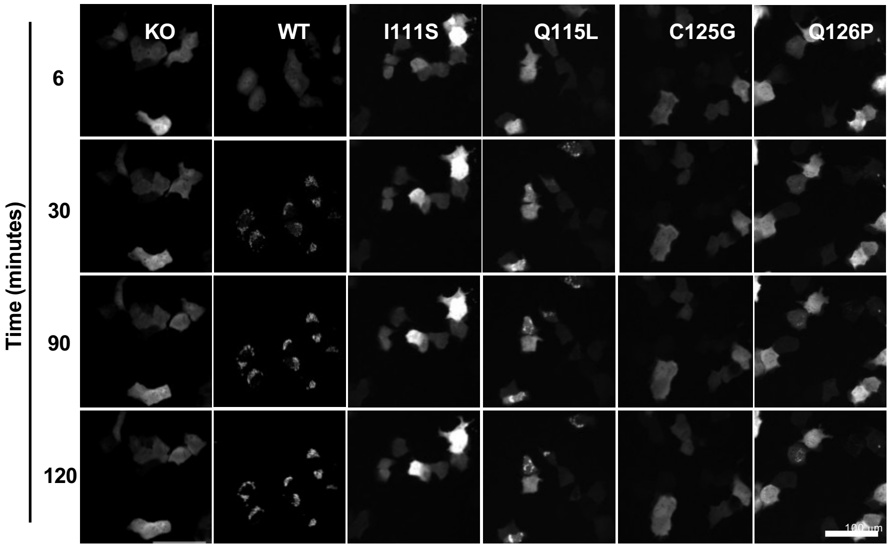
KO
WT
I111S
Q115L
C125G
Q126P
Time (minutes)
6
30
90
120

Figure S8

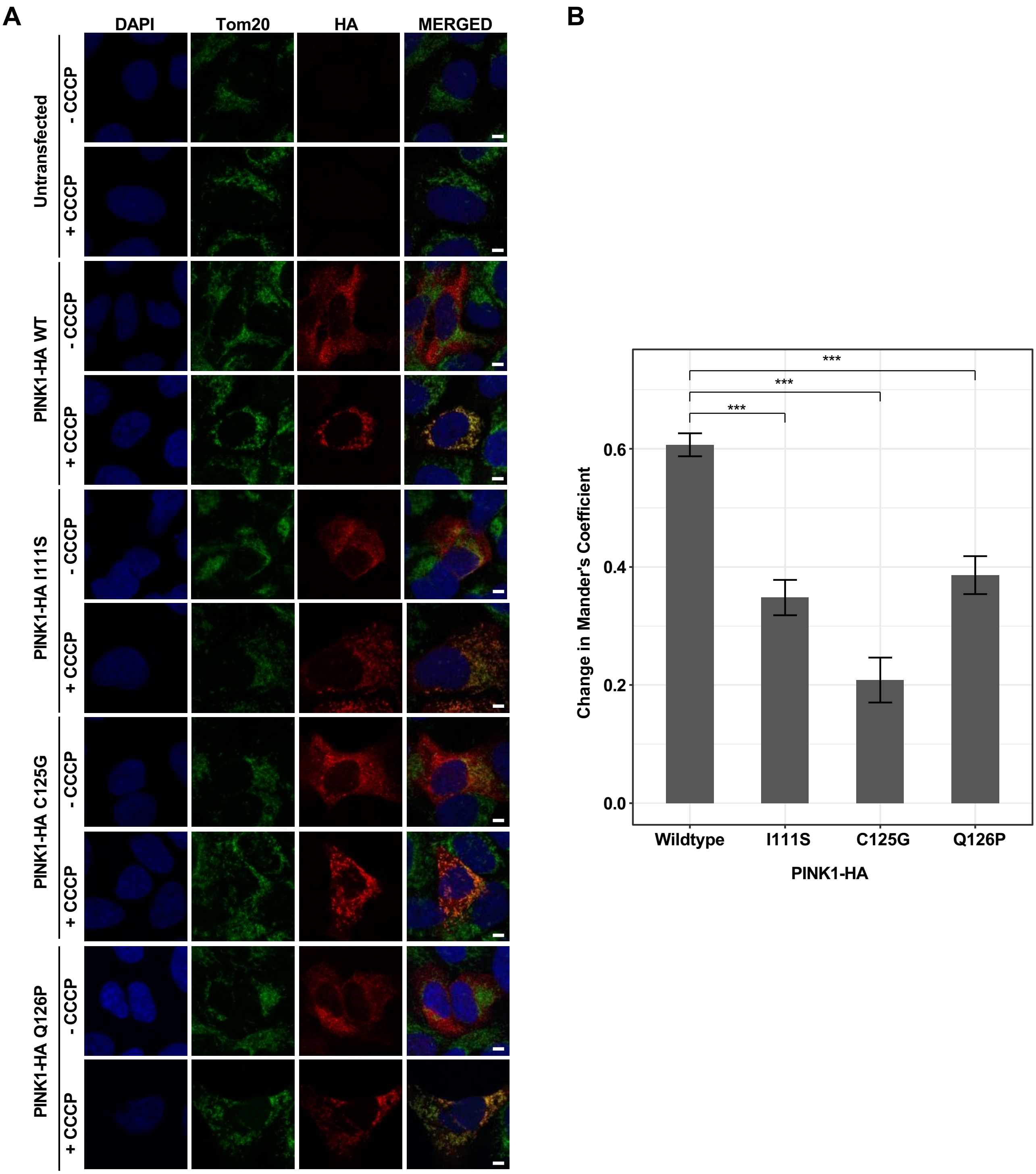
A
DAPI
Tom20
HA
MERGED
Untransfected
- CCCP
+ CCCP
PINK1-HA WT
- CCCP
+ CCCP
PINK1-HA I111S
- CCCP
+ CCCP
PINK1-HA C125G
- CCCP
+ CCCP
PINK1-HA Q126P
- CCCP
+ CCCP
B
Change in Mander's Coefficient
0.0
0.2
0.4
0.6
***
***
***
Wildtype
I111S
C125G
Q126P
PINK1-HA

Figure S9

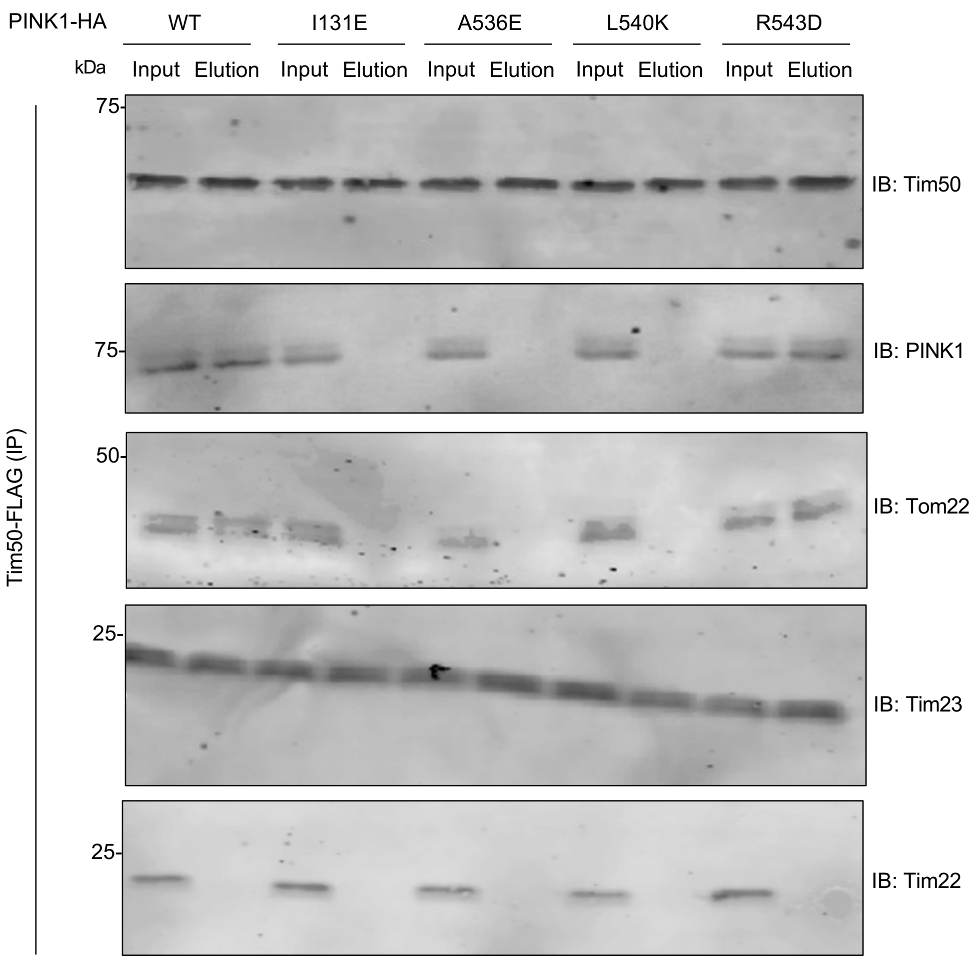
PINK1-HA
WT
I131E
A536E
L540K
R543D
kDa
Input Elution Input Elution Input Elution Input Elution Input Elution
75-
IB: Tim50
75-
IB: PINK1
50-
IB: Tom22
25-
IB: Tim23
25-
IB: Tim22
Tim50-FLAG (IP)
